# Single‐cell and spatial transcriptomics reveal *POSTN* ^+^ cancer‐associated fibroblasts correlated with immune suppression and tumour progression in non‐small cell lung cancer

**DOI:** 10.1002/ctm2.1515

**Published:** 2023-12-19

**Authors:** Chao Chen, Qiang Guo, Yang Liu, Qinghua Hou, Mengying Liao, Yanying Guo, Yupeng Zang, Fei Wang, Huanyu Liu, Xinyu Luan, Yanling Liang, Zhuojue Guan, Yanling Li, Haozhen Liu, Xuan Dong, Xiuqing Zhang, Jixian Liu, Qumiao Xu

**Affiliations:** ^1^ Department of Thoracic Surgery Peking University Shenzhen Hospital Shenzhen Peking University‐The Hong Kong University of Science and Technology Medical Center Shenzhen China; ^2^ BGI Research Hangzhou China; ^3^ BGI Research Shenzhen China; ^4^ College of Life Sciences University of Chinese Academy of Sciences Beijing China; ^5^ Department of Pathology Peking University Shenzhen Hospital Shenzhen China; ^6^ Central Laboratory of Peking University Shenzhen Hospital Shenzhen China; ^7^ Guangdong Provincial Key Laboratory of Human Disease Genomics, Shenzhen Key Laboratory of Genomics BGI Research Shenzhen China

**Keywords:** non‐small cell lung cancer, POSTN, single‐cell RNA sequencing, spatial transcriptomics

## Abstract

**Background:**

Cancer‐associated fibroblasts (CAFs) are potential targets for cancer therapy. Due to the heterogeneity of CAFs, the influence of CAF subpopulations on the progression of lung cancer is still unclear, which impedes the translational advances in targeting CAFs.

**Methods:**

We performed single‐cell RNA sequencing (scRNA‐seq) on tumour, paired tumour‐adjacent, and normal samples from 16 non‐small cell lung cancer (NSCLC) patients. CAF subpopulations were analyzed after integration with published NSCLC scRNA‐seq data. SpaTial enhanced resolution omics‐sequencing (Stereo‐seq) was applied in tumour and tumour‐adjacent samples from seven NSCLC patients to map the architecture of major cell populations in tumour microenvironment (TME). Immunohistochemistry (IHC) and multiplexed IHC (mIHC) were used to validate marker gene expression and the association of CAFs with immune infiltration in TME.

**Results:**

A subcluster of myofibroblastic CAFs, *POSTN* ^+^ CAFs, were significantly enriched in advanced tumours and presented gene expression signatures related to extracellular matrix remodeling, tumour invasion pathways and immune suppression. Stereo‐seq and mIHC demonstrated that *POSTN* ^+^ CAFs were in close localization with *SPP1* ^+^ macrophages and were associated with the exhausted phenotype and lower infiltration of T cells. *POSTN* expression or the abundance of *POSTN* ^+^ CAFs were associated with poor prognosis of NSCLC.

**Conclusions:**

Our study identified a myofibroblastic CAF subpopulation, *POSTN* ^+^ CAFs, which might associate with *SPP1* ^+^ macrophages to promote the formation of desmoplastic architecture and participate in immune suppression. Furthermore, we showed that *POSTN* ^+^ CAFs associated with cancer progression and poor clinical outcomes and may provide new insights on the treatment of NSCLC.

## Graphical abstract

*POSTN* ^+^ CAFs were enriched in advanced NSCLC tumours and presented gene expression signatures related to extracellular matrix remodeling, tumour invasion and immune suppression.

*POSTN* ^+^ CAFs were in close localization with *SPP1* ^+^ macrophages and associated with the exhausted phenotype and lower infiltration of T cells.

*POSTN* expression and *POSTN* ^+^ CAFs were prognostic factors for NSCLC.

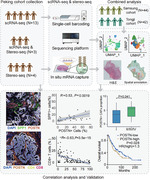

## 1 INTRODUCTION

Lung cancer is one of the leading causes of cancer‐related death, and non‐small cell lung cancer (NSCLC) is the most common subtype of lung cancer.^1^ Immunotherapies, such as immune checkpoint inhibitors (ICIs), have improved the overall survival of NSCLC patients. However, the response rate to ICI therapy is only ∼20%, and there are still a large proportion of patients who are not sensitive to ICI.^2^ Tumour microenvironment (TME), which is composed of tumour cells, stromal cells, and extracellular matrix (ECM), is closely involved in tumour initiation, progression, and resistance to therapies.^3^ Recent studies suggested predictive biomarkers for ICI response based on tumour intrinsic genomic variants and TME phenotypes, for example, positive correlations of tumour mutation burdens, PD‐1/PD‐L1 expression and T cell infiltration with ICI efficacy in NSCLC patients.^4^, ^5^, ^6^, ^7^ To further extend the benefit of ICI treatment, in‐depth analysis of the components and interactive networks in TME is needed to reveal specific cell types that play important roles in immune modulation and tumour progression, which might provide potential targets for combinatorial immunotherapies.^8^

Cancer‐associated fibroblasts (CAFs) represent one of the major stromal cell components in TME, which are activated mesenchymal cells negative for epithelial, endothelial and leukocyte markers, excluding other mesenchymal linages such as pericytes.^9^ They can be derived from different cell origins, such as resident fibroblasts,^10^ bone marrow‐derived mesenchymal stem cells,^11^ macrophages^12^ and pericytes.^13^ Due to phenotypic diversity, classical markers such as α‐smooth muscle actin (αSMA), and fibroblast activation protein alpha (FAP) could identify a proportion of CAFs, while more relevant biomarkers are under research.^14^ Periostin (encoded by *POSTN*) is a matricellular protein overexpressed in cancers including NSCLC compared to normal tissues.^15^, ^16^ CAFs producing periostin promoted the maintenance of cancer stem cell niche, angiogenesis, tumour growth, invasion and metastasis.^17^, ^18^, ^19^, ^20^ Recent studies underscored the essential roles of crosstalk between CAFs and tumour‐infiltrating immune cells on shaping the immune‐suppressive TME.^21^ Tumour‐associated macrophages (TAMs) are the most abundant immune cells in TME and are associated with immunosuppression,^22^ tumour progression^23^ and poor prognosis in various cancer types.^24^, ^25^ Secreted phosphoprotein 1 (*SPP1*) ^+^ macrophages were regarded as a group of universally presented immune‐suppressive TAMs with high activities in angiogenesis and matrix modeling in NSCLC, and the abundance of *SPP1* ^+^ TAMs was associated with a worse clinical outcome.^26^, ^27^ Although the multifaceted roles of periostin on cancer cells were elucidated, the association of *POSTN* ^+^ CAFs with other stroma cells including macrophages, and the influence on T cell infiltration remains to be explored.

With the rapid development of single‐cell RNA sequencing (scRNA‐seq) technology, the complexity and heterogeneity of CAFs have been brought to the spotlight along with essential questions regarding to their functional implications.^28^ Two main CAF subpopulations, myofibroblastic CAFs (myCAFs) with high levels of αSMA and inflammatory CAFs (iCAFs) characterized by high expression of IL‐6 were broadly reported in a variety of solid cancers.^29^, ^30^ It has been described that myCAFs and iCAFs actively contributed to the immune‐suppressive milieu, through ECM remodeling and secreting different growth factors, cytokines, or chemokines, playing pleotropic roles in affecting the response to immunotherapy. They can be further divided into subpopulations in different types and stages of tumours.^28^ Previous studies using scRNA‐seq have identified diverse CAF subpopulations in NSCLC^31^, ^32^; however, detailed phenotypic characterization is still needed to fully reveal the complexity and plasticity of CAFs, especially in the context of spatial structure of TME. The recent advance of spatial RNA‐seq technologies represented a significant inflection point for a deeper analysis of TME architecture from a new dimension.^33^ By combining the scRNA‐seq and spatial RNA‐seq technologies, several studies unraveled the spatial organizations and unique characteristics of CAFs and TAMs in human tissue samples from colorectal cancer, breast cancer, and cervical squamous cell carcinoma,^34^, ^35^, ^36^ while such information is still lacking in lung cancer.

In this study, we classified CAF subpopulations at single‐cell transcriptomic level by integrating our in‐house generated scRNA‐seq data with two published datasets.^31^, ^32^ We identified a myCAF subtype, *POSTN* ^+^ CAFs, associated with the pro‐invasive and immune‐suppressive TME. Using a newly developed, high‐resolution spatial RNA‐seq technology, SpaTial enhanced resolution omics‐sequencing (Stereo‐seq),^37^ we found that *POSTN* ^+^ CAFs clustered around or adjacent to the tumour nests, and were in proximity with *SPP1* ^+^ macrophages, contributing to ECM remodeling and immune suppression. T cells infiltrated less at the tumour regions rich in *POSTN* ^+^ CAFs and showed exhausted phenotypes. The expression of *POSTN* and the abundance of *POSTN* ^+^ CAFs were significantly associated with the clinical stages and poor prognosis of NSCLC. Collectively, our data provided new insights on the characteristics and functions of CAF subtypes in NSCLC and suggested the critical roles of *POSTN* ^+^ CAFs in immune suppression and tumour progression, indicating that they may be promising targets for the treatment of NSCLC.

## 2 RESULTS

### 2.1 Single‐cell transcriptomic profiling of main cell types in the samples of Peking cohort

**FIGURE 1:**
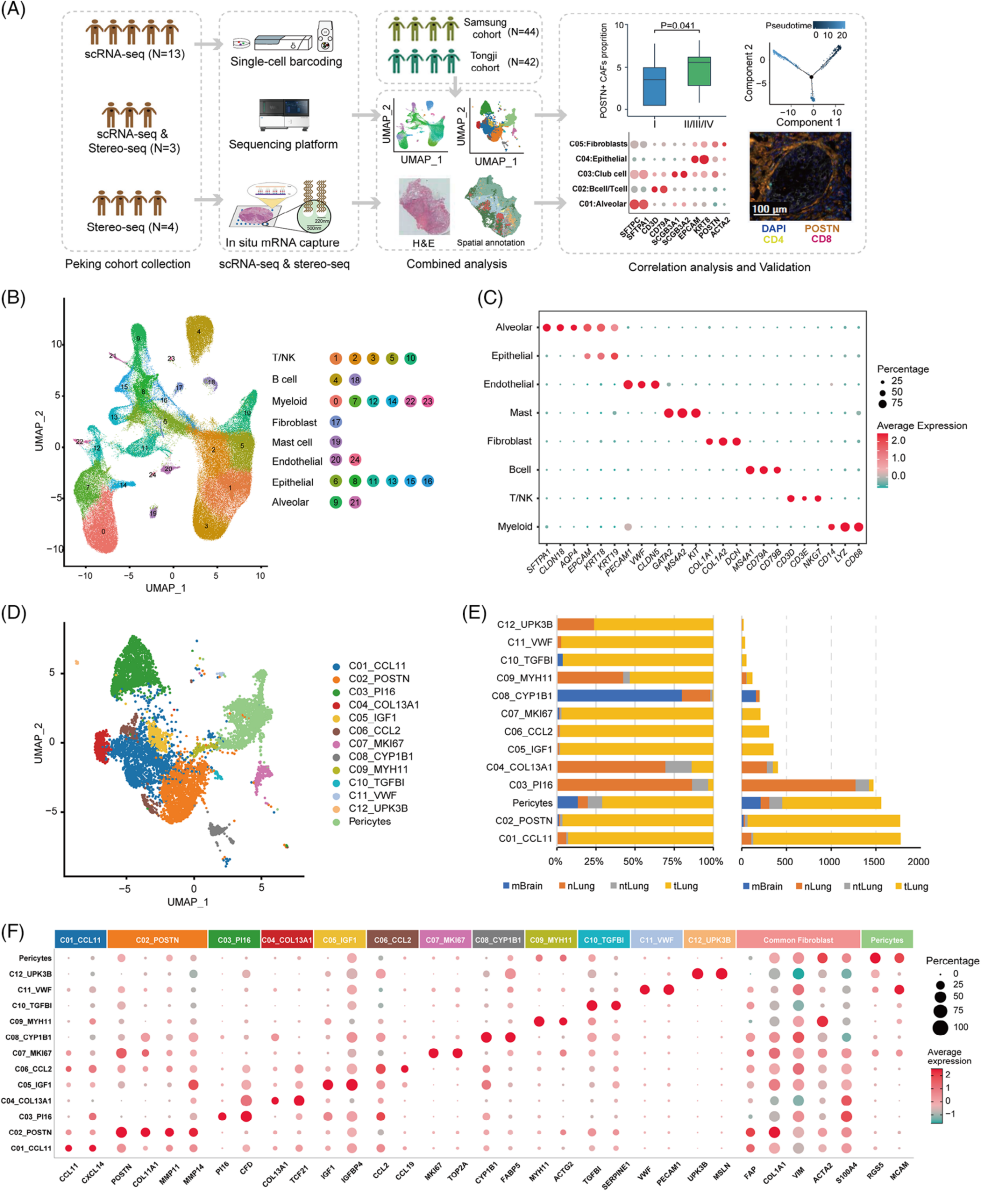
Single‐cell transcriptomic profiling of fibroblasts in non‐small cell lung cancer (NSCLC) samples from three cohorts. (A) Schematic workflow outlining the samples, experimental strategies and bioinformatic analysis for this study. Peking cohort includes samples collected in this study, which were subjected to scRNA‐seq or Stereo‐seq. The scRNA‐seq data from Peking cohort were integrated with the published data of Samsung cohort (*N* = 44) and Tongji cohort (*N* = 42). Bioinformatic analysis of scRNA‐seq and Stereo‐seq data was performed and validated using The Cancer Genome Atlas (TCGA) bulk RNA‐seq data and multiplexed immunohistochemistry (mIHC). N denotes the number of patients. (B) UMAP plot of 162 036 cells from sixteen NSCLC patients in the Peking cohort. Each dot corresponds to one single cell. (C) Dot plot of the average expression of marker genes for eight main cell types. The dot size represents the percentage of cells expressing the genes in each cell type. (D) UMAP plot of fibroblasts from three cohorts coloured by subclusters. (E) Tissue origins of thirteen fibroblast subpopulations represented by the proportion of cells (left) and number of cells (right). (F) The dot plot showing the average expression of signature genes of thirteen fibroblast subpopulations. The dot size indicates the percentage of cells expressing the genes in each cluster. UMAP, Uniform Manifold Approximation and Projection.

We collected samples of tumours (tLung), tumour adjacent tissues (ntLung, tissue within 2 cm adjacent to tumour), and distal normal tissues (nLung, tissue more than 5 cm to tumour) from 20 patients with stage I–IV lung squamous cell carcinoma (LUSC) or lung adenocarcinoma (LUAD) (Table S1). Samples were digested into single‐cell suspensions or OCT‐embedded, followed by scRNA‐seq using BGI‐DNBelab C4 platform^38^ or spatial RNA‐seq through Stereo‐seq technology^37^ (Figure 1A). For most samples used for scRNA‐seq, patient‐matched tLung, ntLung and nLung samples were collected (Table S1). After filtering out the low‐quality cells and putative cell doublets, a total of 162 036 cells were retained for downstream analysis. We then performed principal component analysis (PCA) based on highly variably expressed genes and unsupervised clustering to identify cell types based on their expression patterns. Twenty‐five clusters were classified into distinct cell lineages annotated according to expression patterns of canonical marker genes (Figure 1B,C, Figure S1).^30^, ^31^, ^32^ Thus, eight major cell types were identified, including myeloid cells, T/NK cells, B cells, fibroblasts, mast cells, endothelial cells, epithelial cells, and alveolar cells (Figure 1B,C, Table S2). Cell clustering among sample origins showed no obvious batch effect, and consistent with previous scRNA‐seq studies,^30^, ^32^ differential distribution of cell types was observed across patients (Figure S1B,C).

### 2.2 Identification of diverse fibroblast subpopulations in NSCLC from integrated scRNA‐seq data

Due to difficulties with dissociation of fibroblasts from tissues, a limited number of cells generated in a single study could impose limitations on the thorough analysis of fibroblast subpopulations. To characterize the phenotypic and functional diversity of fibroblasts in NSCLC, we combined the fibroblasts in this study (*N* = 1986) with fibroblasts in another two studies, the Samsung cohort^31^ (*N* = 3499, including nLung, tLung, and brain metastases (mBrain) samples), and the Tongji cohort^32^ (*N* = 4497, including tLung samples), resulting in a total of 9982 fibroblasts (Figure 1A). We then performed reciprocal PCA (RPCA) followed by graph‐based clustering to identify fibroblast subpopulations in NSCLC. After excluding potential low‐quality cells and removing a cluster with high expression of epithelial markers (such as *EPCAM*, *KRT8*, *KRT19*) to distinguish fibroblasts from tumour cells undergoing epithelial‐mesenchymal transition (EMT), 13 subclusters of fibroblasts were retained (Figure 1D, Table S3). Cells from four tissue types (tLung, mBrain, ntLung, nLung) were enriched differently among the fibroblast subclusters (Figure 1E, Figure S2). Distinct from other three tissue types, mBrain samples mainly composed of pericytes, C02_POSTN and C08_CYP1B1. Distribution of fibroblast subpopulations in patients also showed substantial heterogeneity (Figure S2A), while some subclusters, such as C01_CCL11, C02_POSTN and Pericytes were detected in over 50% of patients (Figure S2B). These observations were in accordance with the diverse phenotypes and functions of fibroblasts.^28^, ^39^

We identified differentially expressed genes (DEGs) for each fibroblast subcluster (Figure 1F, Table S4). The pericyte cluster expressed several well‐known pericyte‐associated marker genes, such as *RGS5, CSPG4, ABCC9*, and *KCNJ8*. Clusters C01_CCL11, C05_IGF1, and C06_CCL2 were characterized by high expression of inflammation‐related genes, such as *CCL11, CXCL14*, *CXCL1*, *PLA2G2A*, *APOE*, *C3*, *CCL19*, *CCL5* and *CXCL12*, which were markers for iCAFs as described in other studies.^40^, ^41^ C03_PI16 and C04_COL13A1 were regarded as adventitial and alveolar fibroblasts respectively according to gene expression patterns.^42^ For example, C03_PI16 expressed several marker genes, such as *COL14A1, CFD, GSN* and *PI16*, similar to the major fibroblast subpopulations in normal tissue and early‐stage tumour in a previous study.^31^ The high expression level of *PI16* was associated with a class of universal stem‐like fibroblasts, which can develop into other subpopulations.^42^ C04_COL13A1 expressed several extracellular matrix genes found in the alveolar tissues of mice,^42^ such as *COL13A1*, *TCF21* and *NPNT*, indicating an early differentiated state of this cell cluster. C02_POSTN and C07_MKI67 were distinguished by high expression of *POSTN*, matrix metalloproteinases (MMPs), and other marker genes for myCAFs.^34^, ^43^, ^44^ The matricellular protein periostin encoded by *POSTN* played a key role in forming a fibrotic environment, and promoting cancer proliferation or invasion,^45^ which might partly function via modulating the expression of *MMP2*.^46^

C08‐C12 contained a small number of cells (*N* < 200) and covered less than 15% of patients from three cohorts (Figure 1E, Figure S2), which might represent cells at an intermediate state of differentiation or relatively rare subpopulations. C08_CYP1B1 were mainly composed of cells from the brain metastatic tissue and regarded as the fibroblast‐like cells within the perivascular space of the central nervous system in the Samsung cohort.^31^ C09_MYH11 were found in both nLung and tLung, and represented another subtype of myofibroblasts with high expression of *MYH11, ACTA2, ACTG2* and *TAGLN*, but low expression of *POSTN*. C10‐C12 were enriched in tLung samples. C10_TGFBI displayed high expression of *TGFBI*, a structural homolog of *POSTN*, which was found to promote metastasis of colon cancer by enhancing cell extravasation.^47^ C11_VWF were characterized by co‐expressing marker genes of fibroblasts (*COL1A1, FAP, VIM*, *ACTA2*) and endothelial cells (*VWF, PECAM1, CLDN5, FLT1, RAMP2*), which resembled a subgroup of cells undergoing an EMT transition in gastric cancer.^44^ C12_UPK3B represented a small group of mesothelial cells with high expression of *UPK3B, MSLN, CALB2* and *WT1*.^31^

### 2.3 Phenotypic and gene expression features of fibroblast subpopulations at single‐cell transcriptomic level

**FIGURE 2:**
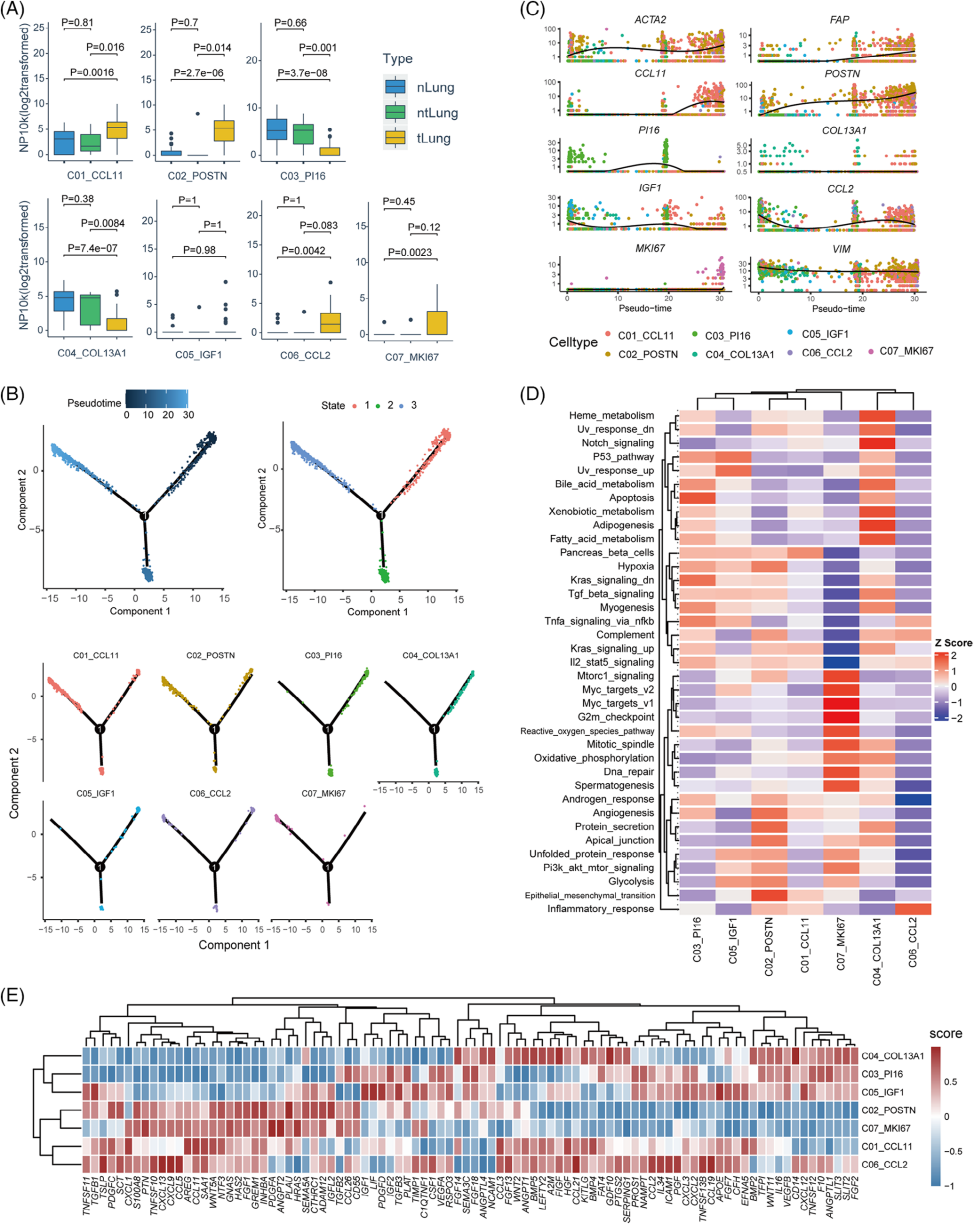
Phenotypic and functional features of fibroblast subpopulations at single‐cell transcriptomic level. (A) Boxplot showing the enrichment of seven fibroblast subpopulations among different tissue types. Each point represents one sample. The *p* value was calculated with the Wilcoxon test. (B) Differentiation trajectories of seven fibroblast subpopulations in a two‐dimensional state‐space defined by Monocle2. Each point corresponds to a single cell. According to the pseudotime, the differentiation paths start from State1. CAF subtypes are enriched at the later stage (state 3). (C) Expression of signature genes of seven fibroblast subpopulations along the pseudo‐time axis using the states in (B) and including all the cells in C01 to C07 clusters. An individual point represents a single cell, and each colour corresponds to a fibroblast subpopulation. The solid black line indicates the pseudo‐time kinetics curves of marker genes, respectively. (D) Heatmap plot showing the activities of seven fibroblast subpopulations in cancer hallmark pathways. (E) Heatmap plot showing ligand expression in seven fibroblast subpopulations.

For the top seven clusters with substantial number of cells (C01‐C07, except for pericytes), we performed statistical analysis using the scRNA‐seq data and the bulk RNA‐seq data of NSCLC from The Cancer Genome Atlas (TCGA). The proportions of C03_PI16 and C04_COL13A1 in ntLung and nLung were higher than that in tLung, representing the normal matrix fibroblasts in lung tissues, while C01_CCL11, C02_POSTN, C06_CCL2 and C07_MKI67 were significantly enriched in tumours (Wilcoxon rank‐sum test, *p* < .05, Figure 2A). We extracted the gene expression matrices of these clusters and calculated their percentages in the tumour and normal samples of TCGA‐NSCLC cohort with the CIBERSORTx algorithm.^48^ Consistently, we found that the proportions of C01_CCL11, C02_POSTN, C06_CCL2 and C07_MKI67 were significantly higher in tumour than normal tissues, both in LUAD and LUSC (Figure S3). Furthermore, pseudo‐time trajectory analysis using Monocle2^49^ showed that C03_PI16 and C04_COL13A1 clustered at the beginning of the differentiation trajectory, and expression of marker genes for normal fibroblasts, such as *ADH1B, CFD, COL14A1* and *GSN* decreased along the pseudo‐time axis (Figure 2B,C, Figure S4). By contrast, C02_POSTN, C06_CCL2, and C07_MKI67 distributed towards the later phase along the differentiation path, and expression of CAF‐related marker genes, for example, *FAP, ACTA2, CCL11, COL8A1*, *CXCL14*, *POSTN, INHBA, COL10A1* and *COL11A1*, also increased along the differentiation trajectory (Figure 2B,C, Figure S4), which was in line with the more differentiated status of CAFs compared to normal fibroblasts.^42^

Gene set variation analysis (GSVA) using cancer hallmark gene sets^50^ was applied to compare the signaling pathway activities among CAF subpopulations (Figure 2D). C01_CCL11 and C02_POSTN shared several similar pathway activities, such as ‘angiogenesis’, ‘EMT’ and ‘IL2‐Stat5 signaling’, suggesting their pro‐invasive roles in TME.^51^ C02_POSTN demonstrated the highest activity in ‘protein secretion’, in accordance with DEGs encoding for various matrix proteins, which implied the essential role of C02 in ECM remodeling. In addition, C02_POSTN, C05_IGF1 and C07_MKI67 had high pathway activities in ‘glycolysis’, ‘unfolded protein response’, and ‘PI3K‐AKT‐mTOR signaling’. C06_CCL2 presented the strongest signal in the ‘inflammatory response’ pathway, consistent with the molecular features for iCAFs. C07_MKI67 up‐regulated multiple cell proliferation pathways, such as ‘DNA repair’, ‘G2M checkpoint’, ‘Mitotic spindle’, therefore regarded as a group of cycling CAFs.

Next, we took a closer look at the ligand expression levels of CAF subpopulations, to infer their roles in regulating anti‐tumour immune responses (Figure 2E). C01_CCL11 and C06_CCL2 were similar in ligand expression patterns, and they might attract eosinophils, basophils, or CCR5^+^ T cells through producing high levels of CCL2, CCL3, CCL11 and CCL21.^52^ The ligands enriched in C02_POSTN and C07_MKI67 included the Transforming Growth Factor‐β (TGF‐β) superfamily and WNT/β‐catenin pathway genes, such as *TGFB1, TGFB2, TGFB3, INHBA* and *WNT5A* suggesting that they might be closely related to the exclusion of T cells and immune evasion.^53^, ^54^ In addition, *CTHRC1* and *ADAM12*, which were highly expressed in C02_POSTN and C07_MKI67 could promote tumour progression, and affect the infiltration of immune cells and polarization of M2 macrophages.^55^, ^56^, ^57^ Since *POSTN* ^+^ CAFs (C02_POSTN and C07_MKI67) might be closely related to the pro‐invasive and immunosuppressive TME, we confirmed the co‐expression of key markers, including periostin (POSTN), COL11A1 and αSMA (ACTA2) by immunohistochemistry (IHC) staining (Figure S5) on two formalin‐fixed paraffin‐embedded (FFPE) NSCLC samples.

### 2.4 *POSTN* ^+^ CAFs and *SPP1* ^+^ macrophages were in proximity and might interact to promote ECM remodeling and immunosuppression

**FIGURE 3:**
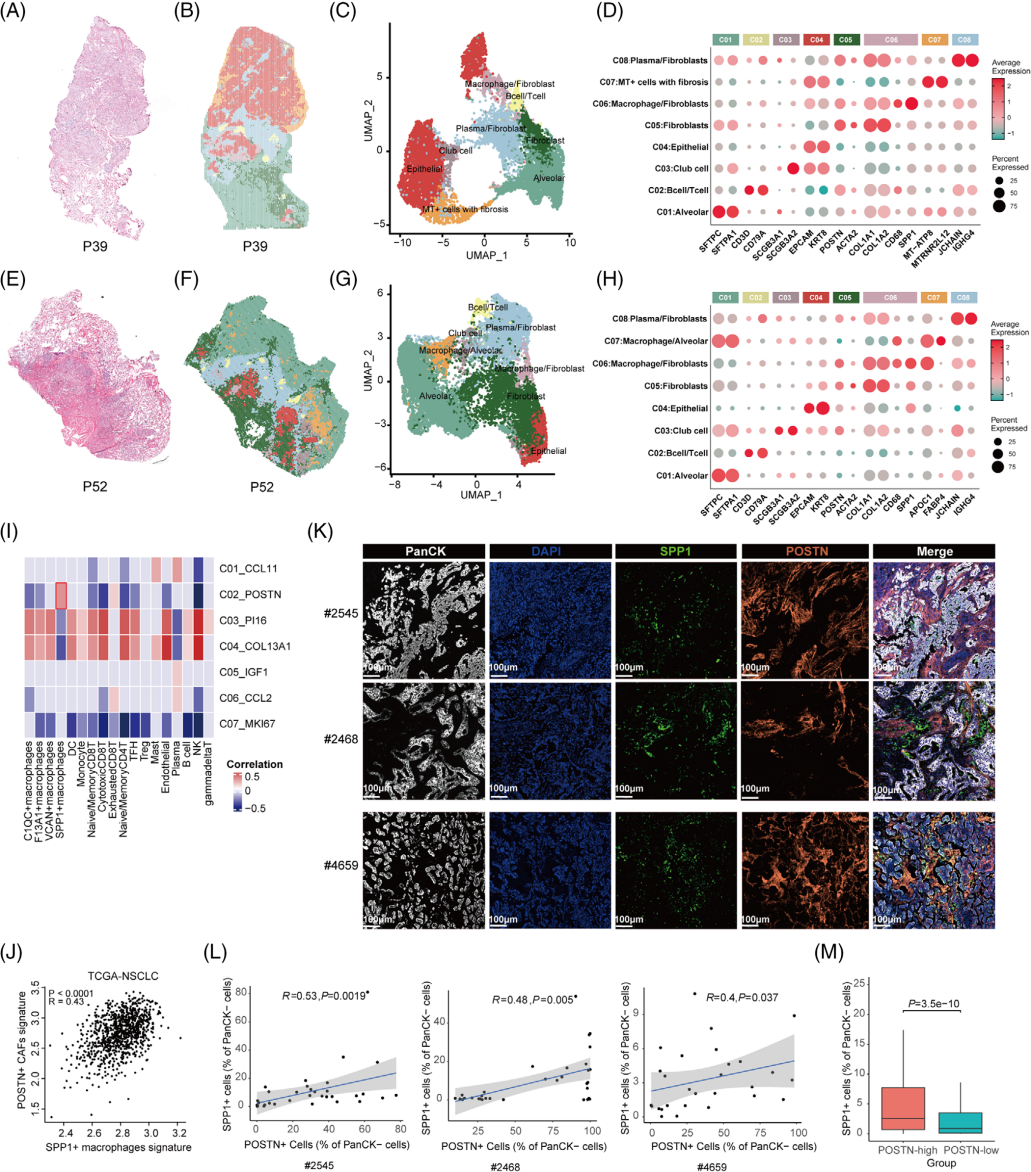
Stereo‐seq reveals spatial proximity of *POSTN* ^+^ cancer‐associated fibroblasts (CAFs) and *SPP1* ^+^ macrophages in non‐small cell lung cancer (NSCLC). (A–H) Hematoxylin and eosin (H&E) staining of the consecutive slides for Stereo‐seq in P39 (A) and P52 (E). Unbiased clustering of Stereo‐seq bins and UMAP plot for the bin clusters for P39 (B and C) and P52 (F and G). Each colour corresponds to an annotated bin cluster. The expression of signature genes across annotated bin clusters for P39 (D) and P52 (H). The dot size represents the percentage of bins expressing the genes in each bin cluster. (I) Heat map depicting the spearman correlation between CAF sub‐populations and other major cell types using scRNA‐seq data of three cohorts. The correlations with *p* values greater than .1 were marked as gray. (J) Spearman correlations of the gene signature scores of the *POSTN* ^+^ CAFs (y‐axis) with those of *SPP1* ^+^ macrophages (x‐axis) using The Cancer Genome Atlas (TCGA)‐NSCLC data. (K) Representative multiplexed immunohistochemistry (mIHC) staining images of tumour cells, POSTN^+^ CAFs, and SPP1^+^ macrophages in formalin‐fixed paraffin‐embedded (FFPE) tumour tissues of three NSCLC patients. PanCK (white), DAPI (blue), SPP1 (green), POSTN (orange), in individual and merged channels are shown. Scale bar, 100 μm. (L) Correlation analysis of SPP1^+^ cells and POSTN^+^ cells based on mIHC staining in FFPE tumour tissues of three NSCLC patients. (M) Proportions of SPP1^+^ cells in POSTN‐high and POSTN‐low regions in FFPE tumour tissues of fifteen NSCLC patients. About 30 regions (including POSTN‐high and POSTN‐low regions, 931 × 698 μm per region) were randomly selected from each tumour sample for Spearman correlation analysis and cell proportion calculation. Wilcoxon test was used to assess statistical significance in M. UMAP, Uniform Manifold Approximation and Projection.

To investigate the spatial localization and potential regulatory roles of CAFs in TME, we performed Stereo‐seq on OCT‐embedded frozen slides of 5 NSCLC specimens and three tumour adjacent tissues (Table S1), and the next consecutive sections were preserved for hematoxylin and eosin (H&E) staining (Figure 3A,E). Stereo‐seq utilized mRNA capture in situ by DNA nanoballs (DNBs) with approximately 220 nm diameter and a center‐to‐center distance of 500 nm.^37^ To decipher the interior architecture of NSCLC samples, we used Seurat to cluster the spatial gene expression of each specimen separately at bin100.^36^ On average, about 1600 genes and about 3500 transcripts were captured by Stereo‐seq at each bin100‐defined unit (100 × 100 DNBs, i.e., ∼50 x 50 μm area, Figure S6). At the resolution of bin100, spatial clustering not only represented cell types, but also reflected the spatial proximity. The spatial clusters were depicted on the tissue section and Uniform Manifold Approximation and Projection (UMAP) (Figure 3B,C,F,G), and annotated using the marker genes consistent with those in scRNA‐seq analysis (Figure 3D,H and Figure S7). The spatial distribution of major cell types corresponded to the pathological annotation based on H&E staining. CAF marker genes, such as *COL1A1*, *COL1A2* and *POSTN*, were expressed at high levels in bin clusters containing fibroblasts (C05: Fibroblasts, C06: Macrophage/Fibroblasts and C08: Plasma/Fibroblasts; Figure 3D,H), suggesting that these clusters were enriched with *POSTN^+^* CAFs. Notably, *CD68* and *SPP1*, which were markers for pro‐angiogenic TAMs with immunosuppressive properties,^26^ were highly expressed in the bin cluster of ‘Macrophage/Fibroblasts’ in two tumours (P39 and P52) (Figure 3D,H), suggesting that *POSTN* ^+^ CAFs and *SPP1^+^* macrophages might be close in spatial positioning in the TME of NSCLC. Using the Robust Cell Type Decomposition (RCTD) algorithm,^58^ we projected cell types based on gene expression matrices from scRNA‐seq data to the spatial transcriptomic map and found that *POSTN* ^+^ CAFs and *SPP1* ^+^ macrophages were enriched in another two tumours (P19 and P47, both stage III) (Figure S8).

To perform correlation analysis of *POSTN* ^+^ CAFs with different immune cell subtypes, we sub‐clustered T/NK cells, B cells, and myeloid cells in the scRNA‐seq data according to canonical markers (Materials and Methods, ‘Cell clustering and annotation using scRNA‐seq data’). T/NK cells were subclustered into cytotoxic CD8^+^ T cells, naive/memory CD8^+^ T cells, exhausted CD8^+^ T cells, regulatory T cells (Tregs), T follicular helper cells (TFH), naive/memory CD4^+^ T cells, gamma delta T cells and NK cells. Myeloid cells were subclustered into dendritic cells (DC), monocytes and macrophages, which were further divided to *SPP1* ^+^ macrophages, *VCAN* ^+^ macrophages, *F13A1* ^+^ macrophages, and *C1QC* ^+^ macrophages (Figures S9–S11). Using signature genes of M1/M2 macrophages,^26^ we found the co‐expression of both M1 and M2 gene signatures in macrophage subsets (Figure S12), consistent with previous studies.^26^, ^59^ *SPP1* ^+^ macrophages showed a higher M2 signature, suggesting that they may function as M2‐like macrophages. The abundance of *POSTN* ^+^ CAFs was positively correlated with *SPP1* ^+^ macrophages (*R* = .36, *p* = .00068), exhausted CD8^+^ T cells (*R* = .20, *p* = .070), and negatively correlated with cytotoxic CD8^+^ T cells (*R* = −.39, *p* = .00027) (Figure 3I). We selected the top10 up‐regulated markers from *POSTN* ^+^ CAFs (top10 marker genes: *POSTN, COL11A1, COL10A1, INHBA, CTHRC1, THBS2, SULF1, COL12A1, PRSS23, FAP*) and *SPP1* ^+^ macrophages (top10 marker genes: *SPP1, RNASE1, A2M, FOLR2, PLA2G7, MARCKS, NPL, ACP2, LILRB4, FAM20C*) respectively and demonstrated that gene signatures for these two cell types were significantly correlated (*p* < .0001, *R* = .43, Figure 3J) in the TCGA‐NSCLC cohort.

To validate the spatial localization of *POSTN* ^+^ CAFs and *SPP1* ^+^ macrophages in the TME of NSCLC, we performed multiplexed IHC (mIHC) using FFPE tumour samples from fifteen NSCLC patients. Representative staining images showed that signals for periostin (POSTN) and osteopontin (SPP1) had similar spatial distribution surrounding tumour nests (Figure 3K). We randomly selected 30 regions on each sample, including POSTN‐high and POSTN‐low tumour regions. Spearman correlation analysis of the percentages of SPP1^+^ cells in PanCK^−^ stromal cells with those of POSTN^+^ cells suggested that they were positively correlated (Figure 3L). We then counted the proportions of SPP1^+^ cells in POSTN‐high and POSTN‐low regions in fifteen patients. The proportions of SPP1^+^ cells in POSTN‐high regions were significantly higher than those in POSTN‐low regions (P = 3.5e‐10, Wilcoxon test, Figure 3M). The results of spatial transcriptomics and mIHC demonstrated significant positive correlation of *POSTN* ^+^ CAFs and *SPP1* ^+^ macrophages, which were consistent with the analysis using scRNA‐seq data and bulk RNA‐seq data (Figure 3I,J), and further suggested the spatial proximity of these two cell types in the TME.

It has been suggested that the interactions between *FAP* ^+^ CAFs and *SPP1* ^+^ macrophages could promote the formation of a desmoplastic TME in colorectal cancer.^34^ To investigate the potential interactions between *POSTN* ^+^ CAFs and *SPP1* ^+^ macrophages in NSCLC, we performed cell‐cell communication analysis with the R package ‘NicheNet’ based on the expression of ligand‐receptor pairs and published interaction databases.^60^ We identified several ligand‐receptor pairs between *POSTN* ^+^ CAFs and *SPP1* ^+^ macrophages, such as *COL4A1*‐*ITGB1*, *TNC*‐integrins (*ITGAX, ITGAV, ITGB1* and *ITGA5*), *TGFB1*/*INHBA‐ACVRL1* and *TGFB1*/*TGFB3*‐*TGFBR1*/*TGFBR2* (Figure S13), which might enhance the attachment and immune‐modulatory activities of *SPP1* ^+^ macrophages. In turn, high ligand activity and expression of *ITGAM*, *SPP1*, *IL1β, TNF* and *TGFB1* in *SPP1* ^+^ macrophages might affect the phenotypes and functions of *POSTN* ^+^ CAFs through regulating various target genes (Figure S14). Taken together, our findings suggested that *POSTN* ^+^ CAFs and *SPP1* ^+^ macrophages presented close localization and might have interactions to promote ECM remodeling and the immunosuppressive TME in NSCLC.

### 2.5 *POSTN* ^+^ CAFs were associated with exhausted phenotypes and lower infiltration of T cells in NSCLC

**FIGURE 4:**
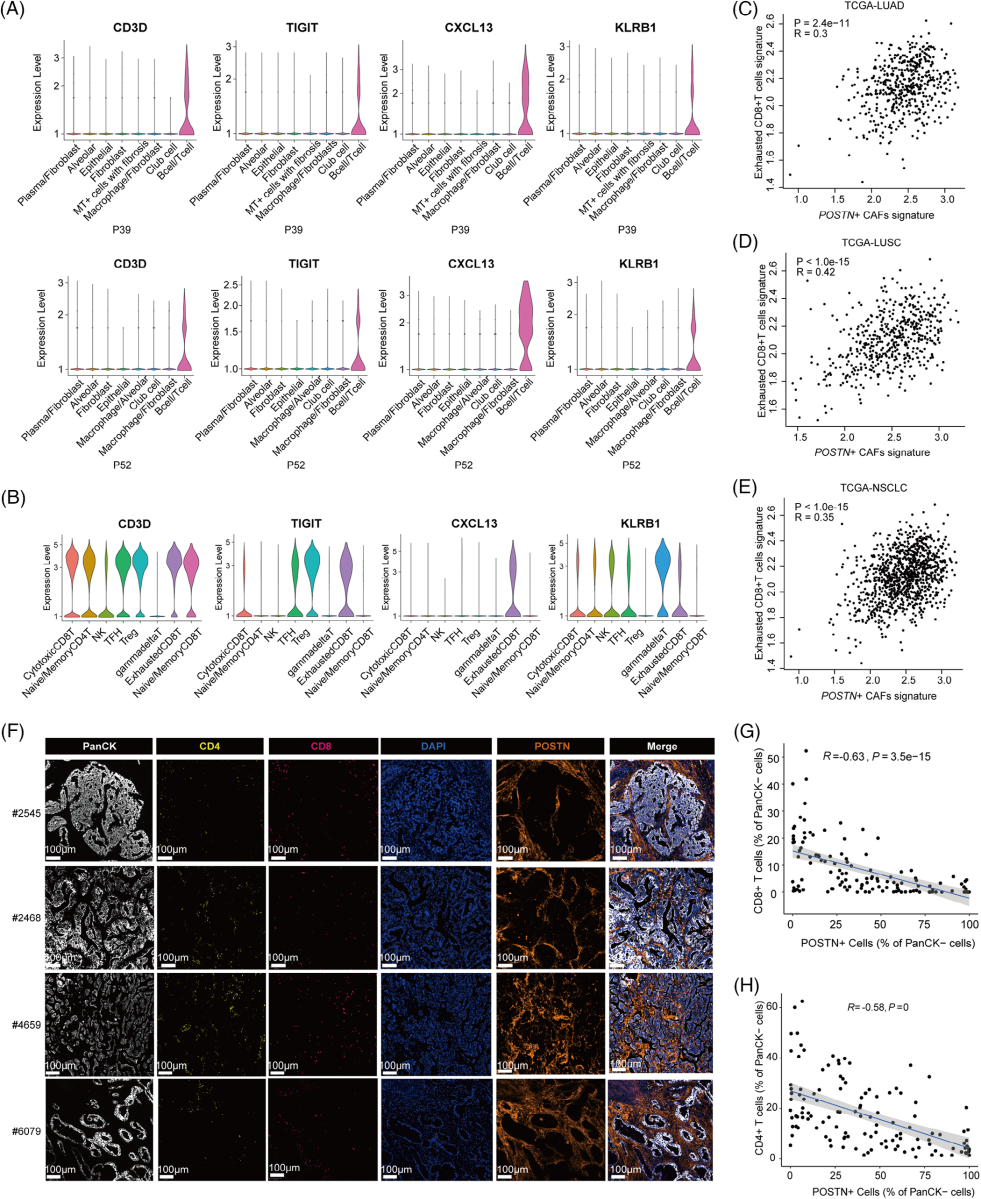
*POSTN* ^+^ cancer‐associated fibroblasts (CAFs) were associated with the exhausted phenotype and lower infiltration of T cells. (A) Violin plots showing expression levels of T cell marker genes across spatial bin clusters in P39 and P52. (B) Violin plots showing marker gene expression in T/NK subclusters based on the scRNA‐seq data of Peking cohort. (C–E) Spearman correlations of gene signatures of exhausted CD8^+^ T cells and *POSTN* ^+^ CAFs in The Cancer Genome Atlas (TCGA)‐LUAD (C), TCGA‐LUSC (D) and TCGA‐non‐small cell lung cancer (NSCLC) (E) samples. (F) Multiplexed immunohistochemistry (mIHC) staining of NSCLC formalin‐fixed paraffin‐embedded (FFPE) samples showing the localization of *POSTN* ^+^ CAFs, T cells and tumour cells. PanCK (white), CD4 (yellow), CD8 (red), DAPI (blue), POSTN (orange), in individual and merged channels are shown. Scale bar, 100 μm. Experiments were performed in tumour samples from four NSCLC patients. (G and H) Spearman correlation analysis of POSTN^+^ cells and CD8^+^ T (G) or CD4^+^ T cells (H) based on tumour regions selected from each sample. LUAD, lung adenocarcinoma; LUSC, lung squamous cell carcinoma.

To further explore the infiltration and functional status of T cells in the presence of *POSTN* ^+^ CAFs, we examined the spatial gene expression in the ‘B cell/T cell’ cluster in P39 and P52 and found that several exhaustion‐associated markers, such as *CXCL13*, *TIGIT* and *KLRB1* ^61^, ^62^ were expressed at significant levels (Figure 4A). Correspondingly, in scRNA‐seq data, these markers were mainly expressed in exhausted CD8^+^ T cells, and partially expressed in Tregs or TFH cells (Figure 4B), but not in other T cell clusters or B cells (Figure S9 and S10). Our results suggested that the T cells in the ‘B cell/T cell’ cluster which located adjacent to *POSTN* ^+^ CAFs mainly displayed exhausted phenotypes. We extracted the gene signatures of exhausted CD8^+^ T cells (top10 marker genes: *CXCL13, DUSP4, TNFRSF9, CTLA4, RBPJ, LAG3, ITGAE, CD82, PHLDA1, TIGIT*) and *POSTN* ^+^ CAFs (top10 marker genes: *POSTN, COL11A1, COL10A1, INHBA, CTHRC1, THBS2, SULF1, COL12A1, PRSS23, FAP*), and calculated the correlation coefficients in the TCGA database. They were significantly correlated in LUAD, LUSC as well as combined NSCLC cohorts (all *p* < .0001, Figure 4C–E).

Furthermore, we used mIHC to depict the localization of *POSTN* ^+^ CAFs and T cells in TME. We found that *POSTN* ^+^ CAFs tended to encircle the tumour nests and infiltrated CD4^+^ T cells and CD8^+^ T cells located mostly in the stromal regions formed by multi‐layers of *POSTN* ^+^ CAFs (Figure 4F). This phenomenon was frequently observed in NSCLC samples we tested. For example, in 10 POSTN‐high tumour regions selected from a sample, seven of 10 (70%) regions had *POSTN* ^+^ CAFs surrounding tumour nests, which might impede the infiltration of T cells, while tumour regions without *POSTN* ^+^ CAFs had higher infiltration of T cells into the tumour bed (Figure S15). To conduct quantitative analysis, we selected ∼30 tumour regions (including POSTN‐high and POSTN‐low regions) on each sample, respectively. The ratios of CD8^+^ T cells and CD4^+^ T cells in PanCK^−^ stromal cells were negatively correlated with the existence of POSTN^+^ cells in the tumour regions (*R* = −.63, *P* = 3.5e−15; *R* = −.58, *p* < .0001; Figure 4G,H), suggesting that *POSTN* ^+^ CAFs were associated with lower infiltration of T cells in NSCLC.

### 2.6 *POSTN* ^+^ CAFs were associated with poorer clinical outcomes of NSCLC

**FIGURE 5:**
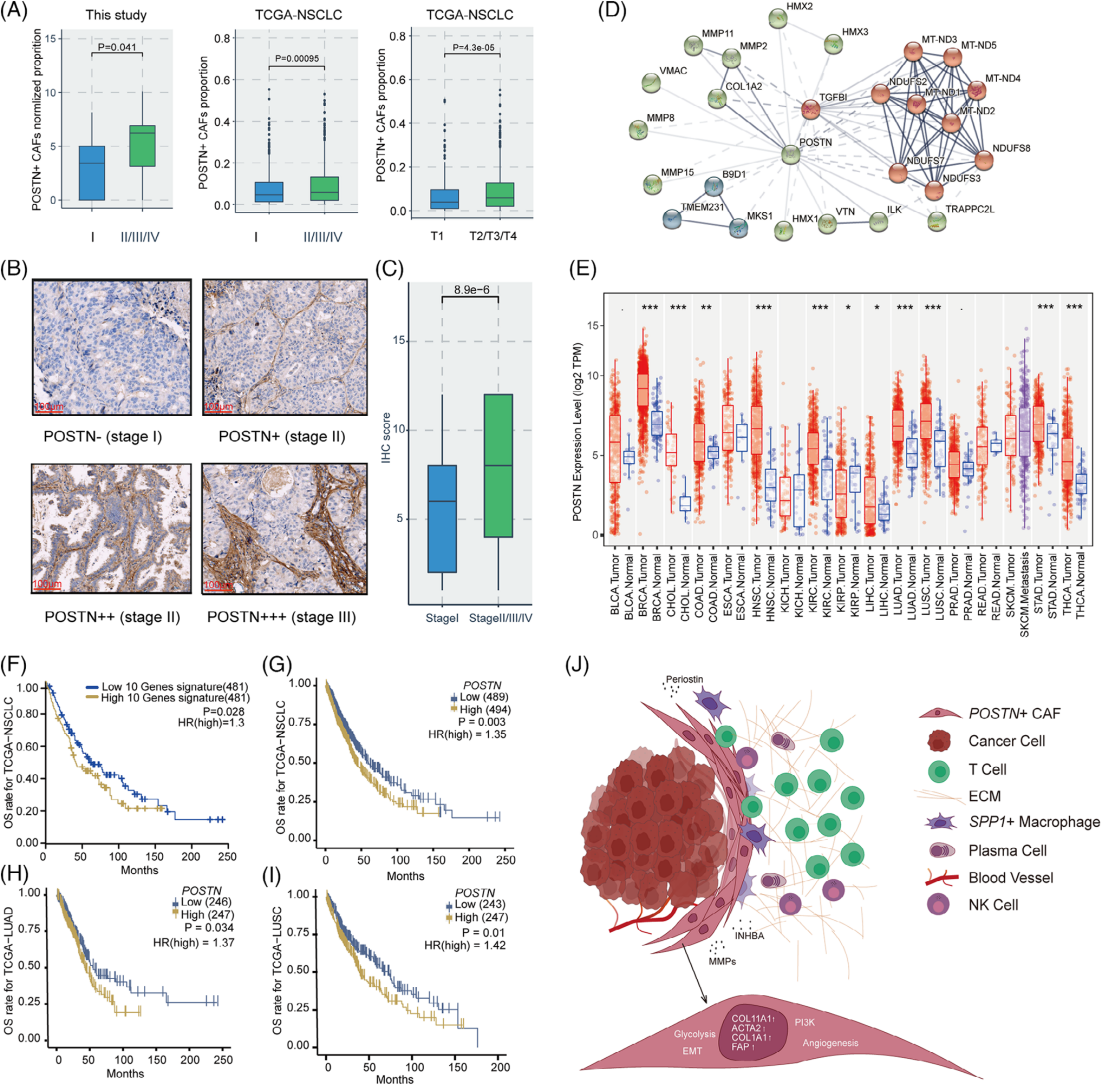
*POSTN* ^+^ cancer‐associated fibroblasts (CAFs) were correlated with cancer progression and poor prognosis. (A) Box plots showing the enrichment of *POSTN* ^+^ CAFs at different clinical stages in three cohorts of this study and the The Cancer Genome Atlas (TCGA)‐non‐small cell lung cancer (NSCLC) cohort, respectively. (B) Immunohistochemistry (IHC) staining of periostin (POSTN) in NSCLC formalin‐fixed paraffin‐embedded (FFPE) samples across different clinical stages. (C) Box plot comparing IHC scores of POSTN between early‐stage NSCLC samples (stage I) and advanced NSCLC samples (stage II–IV). (D) Interaction networks of *POSTN* with other genes based on STRING database analysis. (E) Expression of *POSTN* among pan‐cancer tumour tissues and normal tissues, respectively. Wilcoxon test, ·*p* < .1, *p < .05, **p < .01, ***p < .001. (F‐I) Kaplan–Meier analysis of overall survival rates in TCGA‐NSCLC/LUAD/LUSC cohorts according to expression levels of *POSTN* ^+^ CAFs gene signature or *POSTN*. (J) Schematic illustration of pro‐tumour and immunosuppressive roles of *POSTN* ^+^ CAFs in the tumour microenvironment (TME) of NSCLC. LUAD, lung adenocarcinoma; LUSC, lung squamous cell carcinoma.

Finally, we investigated the presence of *POSTN* ^+^ CAFs in different tumour stages, and their potential association with tumour progression and prognosis. We calculated the relative proportions of *POSTN* ^+^ CAFs in the scRNA‐seq data of this study and showed that *POSTN* ^+^ CAFs were more enriched in advanced tumours (stage II/III/IV) than early‐stage tumours (stage I) (*p* = .041, Figure 5A). By CIBERSORTx analysis in TCGA‐NSCLC samples, we also found that *POSTN* ^+^ CAFs significantly enriched in tumour samples at either advanced TNM stages or T stages (Figure 5A). Furthermore, we measured the levels of POSTN in an independent sample set including 35 archived FFPE NSCLC samples (Figure 5B). We assigned IHC scores to each sample according to the percentage of POSTN^+^ cells as well as staining intensity. The analysis demonstrated that at the protein level, POSTN was significantly related to higher pathological stages (*P* = 8.9e‐6; Figure 5C). STRING database analysis (https://string‐db.org/) showed that *POSTN* acted as a hub gene in the interaction networks connecting with several marker genes of *POSTN* ^+^ CAFs, such as *TGFBI*, *COL1A2*, *MMP2* and *MMP11* (Figure 5D). Besides, *POSTN* upregulation was detected in the tumour tissues of other cancers (Figure 5E), consistent with previous reports on *POSTN* overexpression in a variety of human malignancies,^15^ suggesting that *POSTN* might serve as a common marker for tumour malignant potential.^63^

We plotted the survival curves of NSCLC patients from the TCGA database using the gene signature for *POSTN* ^+^ CAFs or *POSTN* alone. Not only the abundance of *POSTN* ^+^ CAFs predicted unfavorable overall survival in NSCLC patients (*p* = .028 & hazard ratios [HR] = 1.3, Figure 5F), high expression of *POSTN* was also significantly associated with worse survival in NSCLC (*p* = .003 & HR = 1.35, Figure 5G), as well as LUAD and LUSC cohorts (*p* = .034 & HR = 1.37; *p* = .01 & HR = 1.42; Figure 5H,I). The Cox multivariate analysis considering clinical confounding factors (age, sex, smoking, stage, disease type) and signatures of *POSTN* ^+^ CAFs suggested that *POSTN* ^+^ CAFs was an independent prognostic factor for NSCLC (Figure S16).

Together, our results depicted the multi‐faceted tumour‐promoting features of *POSTN* ^+^ CAFs in TME (Figure 5J). *POSTN* ^+^ CAFs presented various pro‐invasive pathway features, including EMT, angiogenesis, glycolysis and protein secretion. ECM factors such as periostin, INHBA, MMPs and TGF‐β produced by *POSTN* ^+^ CAFs could shape the stromal milieu in favour of immune exclusion and tumour growth. Association of *POSTN* ^+^ CAFs with *SPP1* ^+^ macrophages may further promote the formation of desmoplastic and immune‐suppressive TME, contributing to poor prognosis and immunotherapy resistance in NSCLC.

## 3 DISCUSSION

CAFs exert substantial influence on tumour cells and TME, through ECM remodeling, metabolic alterations and secreting factors, which could impact the responses to ICI therapies.^64^, ^65^ Therefore, they are regarded as potential targets for cancer therapy. For example, the clearance of FAP^+^ CAFs by chimeric antigen engineered T cells reduced the desmoplastic stromal structure and tumour vascular density, resulting in significant anti‐tumour effects in human lung cancer xenografts and syngeneic murine pancreatic cancers.^66^ Inhibition of Reactive Oxygen Species‐producing enzyme NOX4 blocked the differentiation of CAFs, thereby promoting the infiltration of CD8^+^ T cells and ICI response in syngeneic murine lung tumour and colorectal tumour models.^67^ However, targeting CAFs in clinics remains challenging, probably due to the plasticity of CAFs and insufficient characterization of specific CAF subtypes in human cancers.^68^

To achieve more comprehensive profiling of fibroblasts and distinguishing specific CAF subtypes as potential targets in NSCLC, we integrated two publicly available scRNA‐seq datasets with the data generated in‐house, resulting in 9982 fibroblast cells from the tumour, tumour adjacent and normal tissues. In line with previous studies,^41^, ^43^ we found that pathways related to collagen production, ECM remodeling, or inflammatory responses were activated in myCAFs or iCAFs. Two myCAF subclusters with high expression of *POSTN*, C02_POSTN and C07_MKI67, were mostly involved in biological functions promoting tumour progression, such as EMT and angiogenesis.^19^, ^20^ They also expressed high levels of ligands in TGFβ and WNT/β‐catenin pathways, which were associated with T cell exclusion and immune suppression.^53^, ^54^ Meanwhile, high expression of *CTHRC1* and *ADAM12* in these subclusters might also contribute to immune exclusion and polarization of M2 macrophages.^55^, ^56^, ^57^ Additionally, correlation analysis based on the scRNA‐seq data demonstrated that C02_POSTN negatively correlated with cytotoxic CD8^+^ T cells or NK cells, and they presented positive correlation with exhausted CD8^+^ T cells and *SPP1* ^+^ macrophages, a cluster of M2‐like macrophages with high expression of M2 gene signatures.^26^ Interestingly, *POSTN^+^* CAFs (C02_POSTN) were identified at substantial amount in multiple mBrain samples, corresponding to the function of periostin in promoting tumour metastasis.^17^, ^18^, ^69^ These results suggested the central roles of *POSTN* ^+^ CAFs in modulating TME to promote tumour progression, immune escape and resistance to immunotherapies, which were also described in other solid tumours.^36^, ^44^

Through spatial transcriptomic analysis, we found that expression of *POSTN*, *COL1A1*, *COL1A2* and *SPP1* was high in the ‘Macrophage/Fibroblast’ cluster, indicating close localization of *POSTN* ^+^ CAFs and *SPP1* ^+^ macrophages. Recent studies have depicted *SPP1* as a prominent marker for TAMs with M2‐like immune‐suppressive functions and poor prognosis in various cancers.^26^, ^59^ The association and interactions of *SPP1* ^+^ macrophages with CAFs promoted the formation of desmoplastic barrier that hindered immune infiltration and limited the efficacies of ICI immunotherapies in hepatocellular carcinoma and colorectal cancer.^34^, ^70^ Previous studies reported that periostin secreted by tumour cells acted as a chemoattractant to enhance the recruitment and polarization of M2 TAMs in glioblastoma and ovarian cancer through periostin‐integrin mediated signaling.^69^, ^71^ We predicted the potential interaction of *SPP1* ^+^ macrophages and *POSTN* ^+^ CAFs through NicheNet.^60^ Similarly, periostin produced by *POSTN* ^+^ CAFs might recruit *SPP1* ^+^ macrophages through integrin receptors, which promoted their spatial proximity. Furthermore, TGF‐β, encoded by *TGFB1* in *SPP1* ^+^ macrophages could bind to the corresponding receptors encoded by *TGFBR1, TGFBR2* and *ACVRL1* on *POSTN* ^+^ CAFs, and osteopontin encoded by *SPP1* might function by interacting with integrin receptors encoded by *ITGB1*, *ITGAV* and *ITGA8* on *POSTN* ^+^ CAFs.^59^ These interactions could result in the activation of a series of target genes encoding collagen or MMPs in *POSTN* ^+^ CAFs. Importantly, these target genes played essential roles in the formation of desmoplastic structure, including extracellular matrix components such as collagens (*COL10A1, COL1A1, COL1A2, COL3A1, COL5A1, COL8A1*), fibronectin (*FN1*), integrins (*ITGA5, ITGB1, ITGB5*), remodeling proteins (*LOX, LOXL1, LOXL2*) and MMPs (*MMP14, TIMP2, TIMP3*).^72^ Additionally, our data of spatial transcriptomics and mIHC showed that T cells were largely excluded from the tumour nests surrounded by *POSTN* ^+^ CAFs. We observed such phenomenon in 13 of 20 (65%) NSCLC samples by mIHC (data not shown). Together, our results suggested that *POSTN* ^+^ CAFs and *SPP1* ^+^ macrophages were closely associated at the tumour stroma, which may be critical in inhibiting tumour immunity and promoting tumour progression, and targeting these cells may enhance the efficacies of ICI immunotherapies in NSCLC.

Furthermore, we demonstrated that *POSTN* expression or the abundance of *POSTN* ^+^ CAFs were significantly associated with advanced tumour stages and poor prognosis in NSCLC. In line with the accumulation of *POSTN* ^+^ CAFs in later‐stage NSCLC, early LUAD featured with ground grass nodules or subsolid nodules were depleted with such CAF subpopulations.^73^, ^74^ In addition, *POSTN* expression could serve as a predictive factor for prognosis, or a biomarker for NSCLC tumour progression.^75^

There are a few limitations in our study that need to be taken into consideration. Firstly, this work provided detailed in silico analysis on CAF subpopulations in NSCLC, while it is also important to assess their functions and interactions with other cell types in experimental settings in future studies. We validated the expression of a few representative markers (*POSTN, ACTA2, COL11A1*) for *POSTN* ^+^ CAFs by IHC, and the gene markers for other CAF subtypes remain to be checked. Secondly, due to the limited samples used for Stereo‐seq, it was difficult to capture all the CAF subtypes in spatial transcriptomic analysis. Last, bulk RNA‐seq data from TCGA database was used in correlation analysis to validate the association of cell types. However, as bulk RNA‐seq data do not contain spatial information, using TCGA cohort can only evaluate the correlation between gene signatures of cell types, which is a limitation for leveraging TCGA data.

In conclusion, we characterized fibroblast subpopulations with diverse phenotypic and gene expression features at the single‐cell transcriptomic level. *POSTN* ^+^ myCAFs were significantly enriched in advanced tumours and presented gene signatures related to pro‐invasion and ECM remodeling. Furthermore, spatial transcriptomic profiling of NSCLC samples combined with mIHC analysis illustrated the close localization of *POSTN* ^+^ CAFs with *SPP1* ^+^ macrophages, and the correlation of *POSTN* ^+^ CAFs with exhausted phenotypes and lower infiltration of T cells. Taken together, our work shed light on the pro‐tumour and immune‐suppression roles of *POSTN* ^+^ CAFs, whose targeting may be beneficial to improve ICI response in NSCLC.

## 4 MATERIALS AND METHODS

### 4.1 Human patient samples

All the NSCLC tumour tissues, tumour adjacent tissues and normal tissues were collected under a protocol approved by Peking University Shenzhen Hospital and BGI Research. Thirty‐five archived FFPE samples were collected retrospectively from the Department of Thoracic Surgery, Peking University Shenzhen Hospital to verify the expression of CAF marker genes.

### 4.2 Tissue processing

The surgical tissues were obtained within 1 h after surgery and divided into two parts. A part of the tissues was stored in MACS Tissue Storage Solution (Miltenyi Biotec, Germany) for up to 24 h at 2−8°C before digestion. The rest were snap‐frozen in OCT compound (ZSGB‐BIO, Beijing) and stored at −80°C for Stereo‐seq and H&E staining.

### 4.3 Preparation of single‐cell suspension from tissues

The fresh tissues were rinsed with pre‐chilled PBS (Thermo Fisher Scientific, USA) twice, mechanically minced, and digested utilizing MACS Human Tumor Dissociation Kit (Miltenyi Biotec, Germany) according to the manufacturer's protocols at 37°C for 15 min. Then the digested tissues were passed through a 70 μm cell strainer (Sartorius, Germany) and centrifuged. The cell pellet was resuspended in PBS containing .04% bovine serum albumin (Merck, Germany) at 1000 cells/μl for scRNA‐seq.

### 4.4 scRNA‐seq using DNBelab C4 system

The DNBelab C4 Single‐Cell Library Prep Set (MGI, Shenzhen) was applied for preparing the scRNA‐seq library. Briefly, single‐cell suspension was firstly encapsulated into single‐cell liquid droplets with barcoded beads followed by cell lysis and mRNA capture. Then emulsion breakage, beads recovery and reverse transcription were performed, followed by the library preparation as previously described.^76^ The constructed libraries were quantified by Qubit ssDNA Assay Kit (Thermo Fisher Scientific, USA) and sequenced by the BGI‐T10 sequencer in the China National Gene Bank (CNGB).

### 4.5 Preparation of stereo‐seq library and sequencing

The STOmics Gene Expression kit S1 (BGI, 1000028493) was utilized according to the standard protocol.^37^ The OCT‐embedded frozen tissue was sectioned into a 10‐μm thick slice, adhered to the Stereo‐seq chip, and incubated at 37°C for 3−4 min. The attached chip was then fixed with methanol at −20°C for 30 min followed by tissue permeabilization, which permit DNBs on the chip surface to capture released mRNA. Afterward, in situ reverse transcription was performed, followed by tissue removal and cDNA releasing. After obtaining the cDNA sequences with spatial barcodes from the chip, they were converted to the cDNA library according to the manufacturer's protocol and quantified by Qubit ssDNA Assay Kit. The sequencing library was sequenced by the BGI‐T10 sequencer in CNGB.

### 4.6 Pre‐processing of scRNA‐seq data

The raw FASTQ files were preprocessed using DNBelab_C_Series_HT_scRNA‐analysis‐software (https://github.com/MGI‐tech‐bioinformatics/DNBelab_C_Series_HT_scRNA‐analysis‐software).

Briefly, the FASTQ raw data were converted to Cell Ranger specific FASTQ files, which were then processed separately using a modified version of Cell Ranger count pipeline. cDNA reads were aligned to GRCH38 human reference using STAR software (v2.5.3).^77^ The mapped reads were then filtered out for valid cell barcodes and unique molecular identifiers to generate gene‐cell matrices for downstream analysis.

### 4.7 Cell clustering and annotation using scRNA‐seq data

Cell clustering was conducted by Seurat (v4.0.6)^78^ package in RStudio. Genes expressed in less than three cells were filtered out, and low‐quality cells were filtered with parameter ‘nFeature_RNA > 300 & nFeature_RNA < 10 000 & nCount_RNA < 25 000 & nCount_RNA > 1000 & percent.mt < 15’. The libraries from the same sample were merged. To deal with the batch effect, the ‘NormalizeData’ and ‘FindVariableGene’ functions were performed respectively for each sample. The potential doublets were further filtered with the ‘DoubletFinder’ package with default parameters. After that, these samples were integrated using the ‘FindIntergrationAnchors’ and ‘IntegrateData’ functions with dims parameter set to 30. The batch effect was checked if the cells were separately distributed with the ‘DimPlot’ function. Then, the integrated data were scaled to calculate the PCA. The first 30 principal components (PCs) were used to construct the SNN network, and the graph‐based clustering method Louvain algorithm was used to identify the cell clusters with a resolution of .6. Finally, UMAP was used to visualize the clustering results in two‐dimensional space.

To annotate each cluster as a specific cell type, we used well‐known canonical markers, dot plots, and violin plots to annotate cell types.^31^The following genes were used for cell type annotation: *CD3D, CD3E, NKG7* (T/NK cells); *CD79A, CD79B, MS4A1* (B cells); *CD14, LYZ* (Myeloid cells); *GATA2, MS4A2, KIT* (Mast cells); *VWF, PECAM1, CLDN5* (Endothelial cells); *COL1A1, COL1A2, DCN* (Fibroblasts); *EPCAM*, *KRT18*, *KER19* (Epithelial cells); *EPCAM*, *CLDN18*, *AQP4* (Alveolar cells).

We further sub‐clustered T/NK cells, myeloid cells and fibroblasts individually. Within the T/NK lineage, we used the following markers for subtype identification: CD8^+^ T (*CD8A*, *CD8B*), CD4+ T (CD4, *IL7R*), naive/memory cells (*CD44*, *CCR7*), cytotoxic T cells (*GZMB*, *PRF1*, *GZMH*, *GNLY*), exhausted T cells (*LAYN*, *HAVCR2*, *PDCD1*, *CTLA4*, *CXCL13*), Tregs (*FOXP3*, *IL2RA*), gamma delta T cells (*TRDC*, *TRGC2*, *TRGC1*) and NK cells (*NKG7, KLRD1, KLRF1*). For the myeloid clusters, four macrophages were identified, including *SPP1* ^+^ macrophages, *VCAN* ^+^ macrophages, *F13A1* ^+^ macrophages and *C1QC* ^+^ macrophages. *SPP1* ^+^ macrophages were positive for markers *SPP1* and *CD163*. Other myeloid cell types were confirmed by specific marker genes including classical monocytes (*CD14, LYZ, FCN1*), and DCs (*CD1C, CLEC10A, LAMP3*).

For fibroblasts sub‐clustering, fibroblasts from this study and other two cohorts, the Samsung cohort (*N* = 3499, including nLung, tLung, and brain metastases samples), and the Tongji cohort (*N* = 4497, including tLung samples), were integrated using ‘FindIntergrationAnchors’ and ‘IntegrateData’ functions. Within fibroblasts (*DCN, COL1A1* and *COL1A2*), *RGS5, ABCC9, KCNJ8*, and *CSPG4* were used to mark the pericytes. The subpopulations of fibroblasts are listed in Table S3.

### 4.8 Cell developmental trajectory

We applied the Monocle2^49^ to determine the pseudo‐time differentiation of diverse fibroblasts populations. We first used the RNA counts of fibroblasts clusters to create a CellDataSet object with parameter ‘expressionFamily = negbinomial.size’ following the Monocle2 tutorial. Cells within the selected fibroblast subpopulations and genes expressed in more than 10 cells were included from subsequent analyses. We used the ‘differentialGeneTest’ function to derive DEGs from each cluster and genes with a *q*‐value < 1e‐10 were used to order the cells in pseudo‐time analysis. Then the cell differentiation trajectory was inferred with the default parameters of Monocle2 after dimension reduction and cell ordering.

### 4.9 DEG analysis and pathway enrichment

To find the marker genes of each fibroblast subpopulation, we performed DEG analysis using the ‘FindAllMarkers’ function in Seurat package with the parameter ‘min.pct = .25, logfc.threshold = .25’. To find the function of marker genes, we used the function compareCluster (fun = ‘enrichGO’, pvalueCutoff = .05, OrgDb = ‘org.Hs.eg.db’) and compareCluster (fun = ‘enricher’, pvalueCutoff = .05, pAdjustMethod = ‘BH’) of R package clusterProfiler (v.4.4.4).

We applied GSVA (version 1.44.5) to assign pathway activity estimates to each fibroblast subpopulation. Cancer hallmark gene sets from Molecular Signatures Database (MSigDB v7.5.1) were used as the input gene sets.

### 4.10 Processing of stereo‐seq data and annotation of bin clusters

The Stereo‐seq data were processed in the same procedure as previous work.^79^ The Stereo‐chip was covered with DNBs to capture spatial transcriptome information of the tissue. We defined 1 × 1 DNB as bin 1, and treated bin100 (100 × 100 DNB) as the basic analysis unit. The raw FASTQ files were mapped to the human genome (hg38), and regions were lassoed out based on H&E staining to remove background noise signals for downstream analysis. After quality control, the lassoed files were processed using the R package Seurat (v4.0.6, https://github.com/satijalab/seurat) to carry out data normalization, scaling, and bin clustering.

The marker genes used in annotation of spatial clusters were consistent with those in scRNA‐seq for cell type annotation. At resolution of bin100, which equals ∼50 x 50 μm area, each bin contained about 20–30 cells. In some bins, markers of two or more cell types were grouped in the same bin, so some bin clusters were annotated as mixed cell types, such as ‘Macrophage/Fibroblast’.

### 4.11 Spatial deconvolution of cell types

Spatial deconvolution of cell types at bin100 was calculated from scRNA‐seq data using RCTD algorithm via R package spacexr‐2.0.0.^80^ For each slice with Stereo‐seq data, matrices of raw counts and spatial coordinates were used to construct the SpatialRNA object in RCTD. Raw counts and annotation of integrated fibroblast cells were used in default parameters as scRNA‐seq reference. Then the reference and SpatialRNA objects were fed into RCTD main function in full mode. Matrices of normalized bin‐cell type probabilities were visualized on each spatial slice and exported for further analysis.

### 4.12 Assessment of the abundances of cell types in TCGA‐NSCLC dataset

The online tool CIBERSORTx (https://cibersortx.stanford.edu/) was used to estimate the abundances of different cell types in TCGA‐NSCLC dataset. The abundance was defined as the number of cells of a particular type divided by the total number of cells in a sample. Briefly, gene expression data from scRNA‐seq in this study, which represented a bulk admixture of different cell types was used to build the signature matrix files for cell types of interest. Subsequently, the obtained gene‐count matrices were used as the input for CIBERSORTx and the proportions of cell types of interest in the bulk TCGA transcriptome data can be evaluated by deconvolving the bulk data.

### 4.13 Cell–cell communication analysis

NicheNet was used to infer the interactions between *POSTN* ^+^ CAFs and *SPP1* ^+^ macrophages.^60^ For ligand and receptor interactions, genes expressed in more than 10% cells of each cluster were considered. Top 15−20 ligands and top 200 receptor targets from DEGs of ‘sender cells’ and ‘receiver cells’, were extracted for paired ligand‐receptor activity analysis. Receiver cells in normal tissues were used as reference cells, and ‘FindMarkers’ method was used to detect DEGs.

### 4.14 IHC staining

Briefly, 3.5‐μm thick FFPE slides were firstly dewaxed, hydrated and washed with flowing water. The antigen retrieval process was then performed with citric acid at 95−100°C for 20 min, cooled down at room temperature for 10 min, and rinsed with water. After that, the tissue area was circled by a PAP pen (MXB biotechnologies, Fuzhou) to ensure even and adequate antibody incubation. The slides were then rinsed with PBS and diluted hydrogen peroxide was applied to quench the endogenous peroxidase activity. Afterwards, the slides were incubated with the primary antibody at 4°C overnight. For each sample, consecutive slides were incubated with anti‐periostin (PA5‐34641, Thermo Fisher Scientific, 1:200 dilution), anti‐COL11A1 (PA5‐115040, Thermo Fisher Scientific, 1:250), and anti‐αSMA (14‐9760‐82, Thermo Fisher Scientific, 1:500), respectively. On the next day, horseradish peroxidase (HRP)‐multimer cocktail (Dartmon, Inc., Shenzhen) and DAB staining was applied. The DAB staining was terminated with water rinsing and hematoxylin counterstaining was performed. Finally, the slides were mounted and processed for imaging. The IHC staining process for frozen slides was similar to FFPE samples, omitting the antigen retrieval step. The IHC scores of POSTN in Figure 5B,C were calculated by multiplying POSTN^+^ % score (0%–5% = 0, 6%−25% = 1, 26%−50% = 2, 51%−75% = 3, > 75% = 4) and staining intensity score (negative = 0, weak = 1, moderate = 2, strong = 3) estimated by a pathologist.

### 4.15 mIHC staining

To detect the spatial locations of *POSTN* ^+^ CAFs (POSTN), *SPP1* ^+^ macrophages (SPP1), T cells (CD4, CD8) and epithelial cells (PanCK), mIHC was performed using BOND RX Fully Automated Research Stainer (Leica, Germany). Briefly, 3.5‐μm thick FFPE tissue sections were deparaffinized and washed in TBST buffer, and then transferred to preheated citrate solution (95°C) for heat‐induced epitope retrieval using a microwave set at 20% of maximum power for 20 min. Slides were stained with the following antibodies/fluorescent dyes, in order: Alexa Fluor 647‐conjugated anti‐CD8 antibody (ab305048, Abcam, 1:100), anti‐osteopontin antibody (ab63856, Abcam, 1:200)/TSA 520, anti‐periostin (hpa012306, Merck)/TSA 620, anti‐CD4 antibody (ab133616, Abcam,1:500) /TSA 570 and anti‐panCytokeratin antibody (4545S, Cell Signaling Technology, 1:400) /TSA 780. Except for fluorescently labeled anti‐CD8 antibody, slides were stained at each cycle using anti‐rabbit/mouse HRP‐conjugated secondary antibody (ARH1001EA, Akoyabio, USA) for 10 min, then reacted with corresponding Tyramide substrates (AXT37100041, Alphaxbio, Beijing). Each slide was then treated with two drops of DAPI (FP1490, Akoyabio), washed in distilled water and manually coverslipped. Slides were air dried and scanned with Vectra Polaris tissue imaging system (Akoya Biosciences, USA). Images were analyzed using Indica Halo software (Indica Labs, USA).

### 4.16 Survival analysis

Survival analysis of *POSTN* ^+^ CAFs for Figure 5F was performed using the online web tool GEPIA2^81^ (http://gepia2.cancer‐pku.cn/#survival). In brief, the top10 signature genes of *POSTN* ^+^ CAFs was tested, and median value was set as cut‐off. Then HRs were calculated using Cox proportional hazards models with 95% confidence intervals reported, and Kaplan–Meier survival curves were modeled using ‘survfit’ functions. The survival analysis of *POSTN* expression for Figure 5G–I was performed using CAMOIP software (http://camoip.net), and median value was set as cut‐off to divide the samples as POSTN‐high and POSTN‐low subgroups. Kaplan–Meier survival curves were compared by a two‐sided rank sum test.

### 4.17 TCGA database analysis

TCGA NSCLC cohort (including LUAD and LUSC) was used to verify the correlation of *POSTN* ^+^ CAFs and *SPP1* ^+^ macrophages signatures (Figure 3J), correlation of *POSTN* ^+^ CAFs and exhausted CD8^+^ T cells signatures (Figure 4C–E), and to conduct survival analysis (Figure 5F–I). Besides, as previously mentioned, we used the CIBERSORTx (https://cibersortx.stanford.edu/) to evaluate the abundance of *POSTN* ^+^ CAFs in different stages of lung cancer using TCGA‐NSCLC cohort.

## AUTHOR CONTRIBUTIONS

Qumiao Xu and Jixian Liu designed and provided financial support for the study. Qiang Guo, Qinghua Hou, Yanying Guo, Huanyu Liu, Zhuojue Guan, Yanling Li, Yanling Liang, and Mengying Liao collected samples and performed experiments. Chao Chen, Yang Liu, Yupeng Zang, Haozhen Liu, and Xinyu Luan performed data analysis. Xuan Dong and Xiuqing Zhang provided experimental materials and platforms. Chao Chen and Qumiao Xu wrote the manuscript. Chao Chen, Qumiao Xu, and Fei Wang edited the manuscript.

## CONFLICT OF INTEREST STATEMENT

The authors declare that they have no competing interests.

## FUNDING INFORMATION

Guangdong Basic and Applied Basic Research Foundation, Grant Number: 2022A1515111138; Guangdong Provincial Key Laboratory of Human Disease Genomics, Grant Number: 2020B1212070028; UMHS‐PUHSC Joint Institute Project, Grant Number: 2019020(PUSH)‐r1); Open Fund Project of BGI‐Shenzhen, Grant Number: BGIRSZ20200003.

## ETHICS STATEMENT

The donors of fresh surgical tissues included in this study have all provided informed consent before enrolling in this project. This study was approved by the Research Ethics Committee of Peking University Shenzhen Hospital and was conducted according to the guidelines of the local law.

## Supporting information

Supplementary Information

Supplementary Information

## Data Availability

The raw scRNA‐seq data and spatial RNA‐seq data in this study have been deposited at China National Center for Bioinformation (CNCB) database (https://www.cncb.ac.cn/?lang = en) under accession code OMIX002370 (scRNA‐seq) and OMIX002367 (spatial RNA‐seq) and deposited into CNGB Sequence Archive (CNSA) of China National GeneBank database (CNGBdb) with accession number CNP0003361. Other data reported in this study are available in the supplementary materials or by contacting the corresponding author.
