# Supplementary material for: Single‐cell and spatial transcriptomics reveal POSTN + cancer‐associated fibroblasts correlated with immune suppression and tumour progression in non‐small cell lung cancer
Source: Clin Transl Med. 2023 Dec 19;13(12):e1515. doi: 10.1002/ctm2.1515 (PMC10731139; doi:10.1002/ctm2.1515)
Supplement: Supplementary file 1 — Supplementary Information [file CTM2-13-e1515-s001.pptx]

## Slide 1
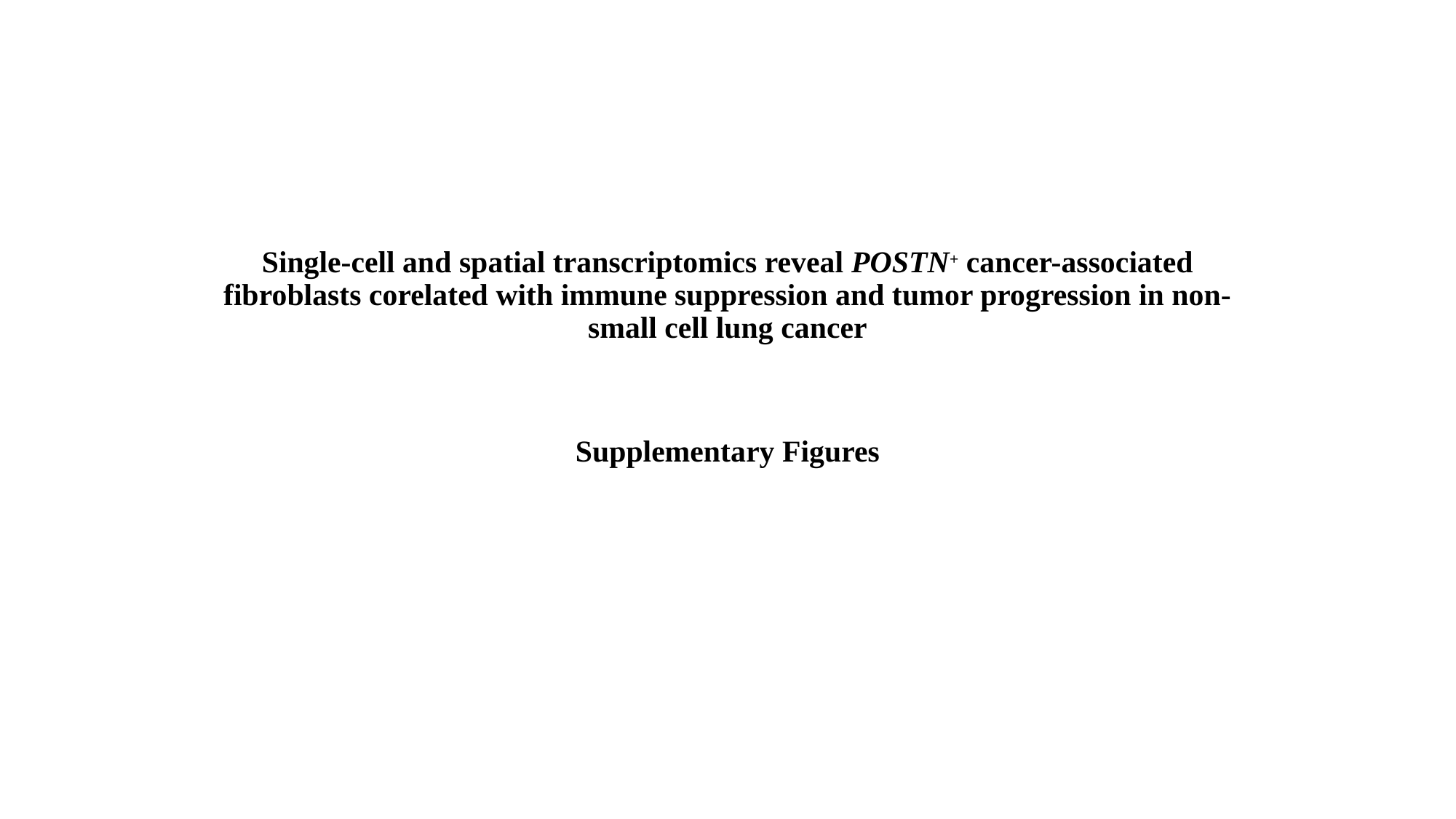

# Single-cell and spatial transcriptomics reveal POSTN+ cancer-associated fibroblasts corelated with immune suppression and tumor progression in non-small cell lung cancer
Supplementary Figures

## Slide 2
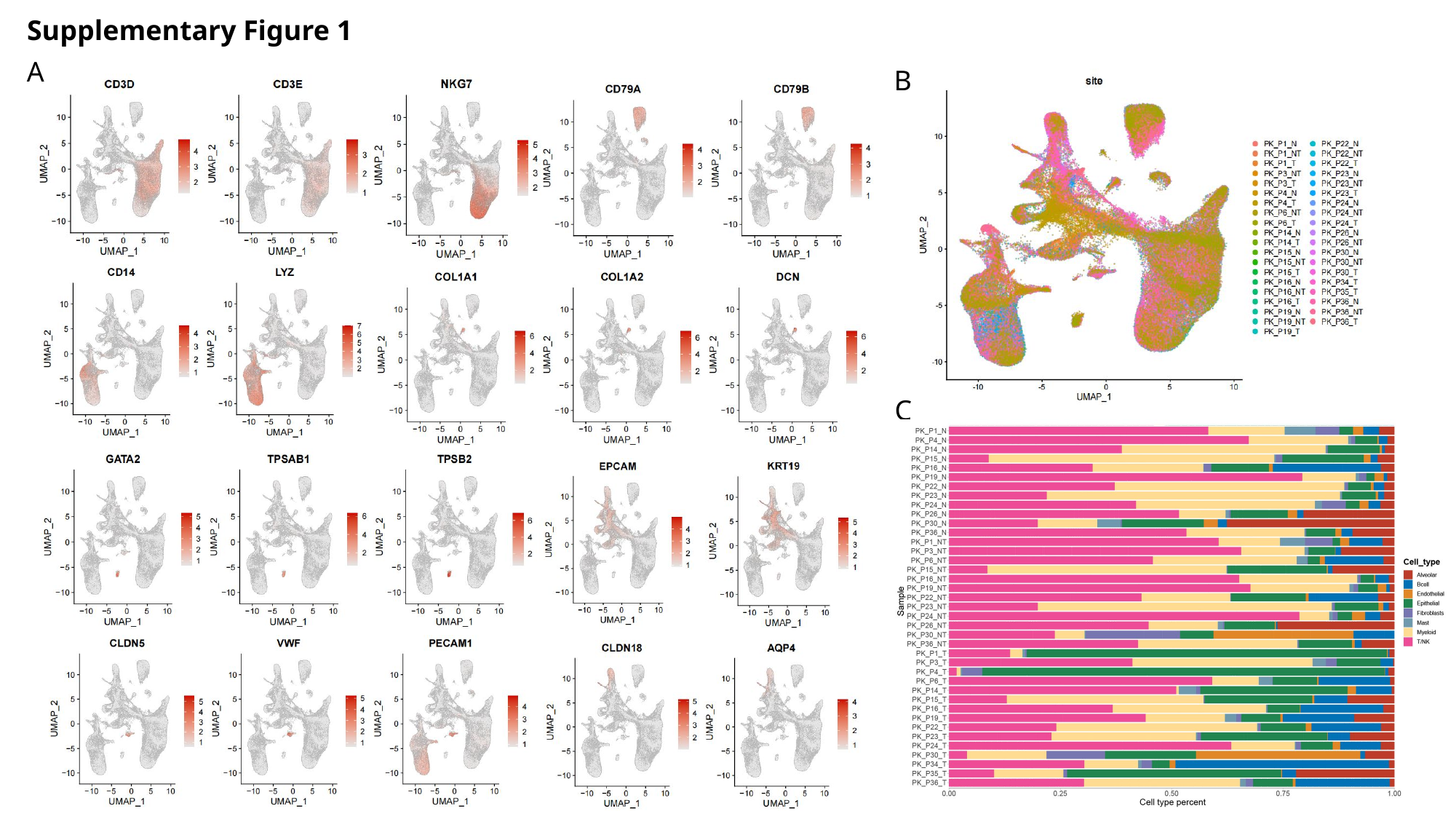

Supplementary Figure 1
A
B
C

## Slide 3
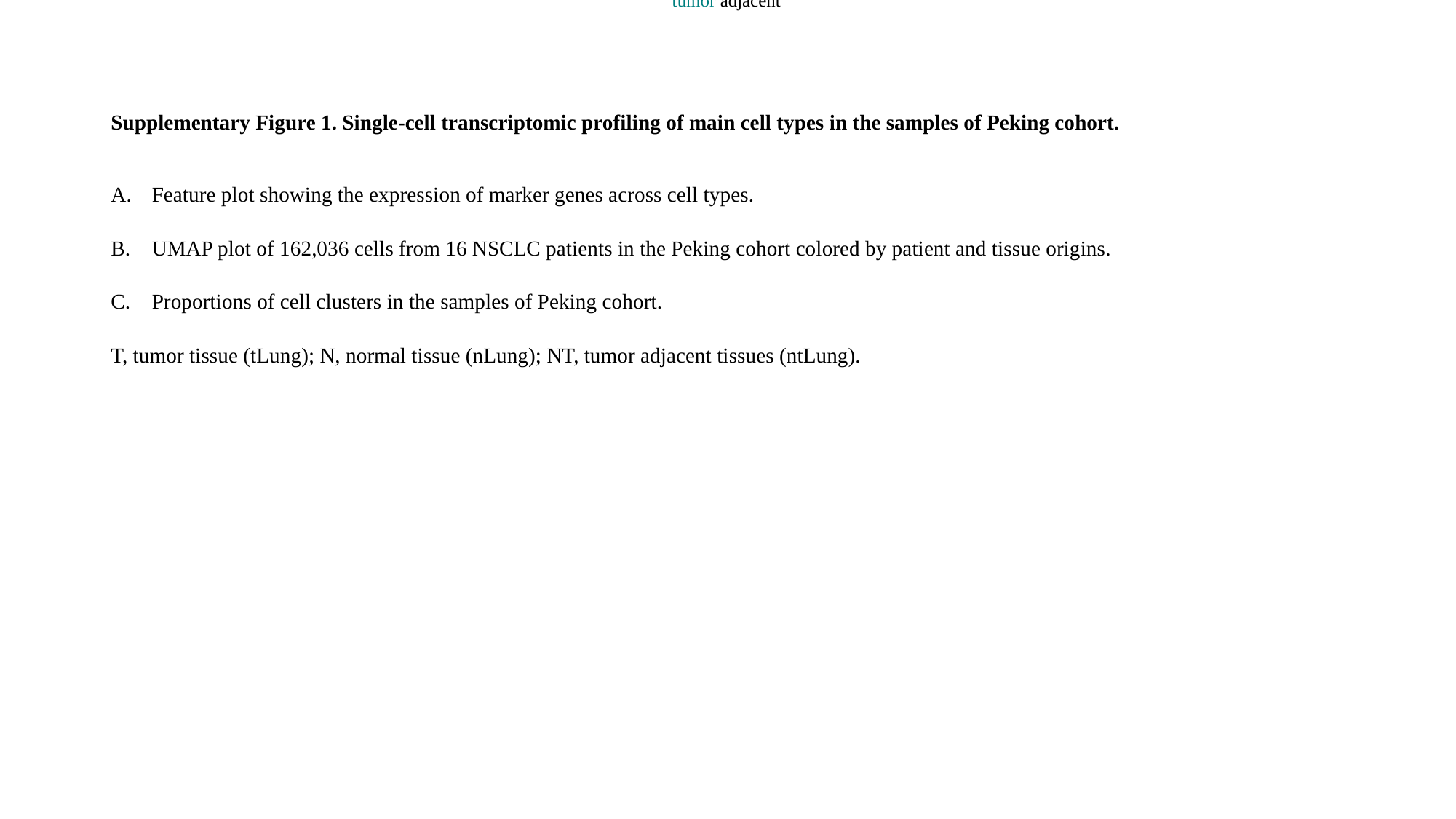

tumor adjacent
# Supplementary Figure 1. Single-cell transcriptomic profiling of main cell types in the samples of Peking cohort.
Feature plot showing the expression of marker genes across cell types.
UMAP plot of 162,036 cells from 16 NSCLC patients in the Peking cohort colored by patient and tissue origins.
Proportions of cell clusters in the samples of Peking cohort.
T, tumor tissue (tLung); N, normal tissue (nLung); NT, tumor adjacent tissues (ntLung).

## Slide 4
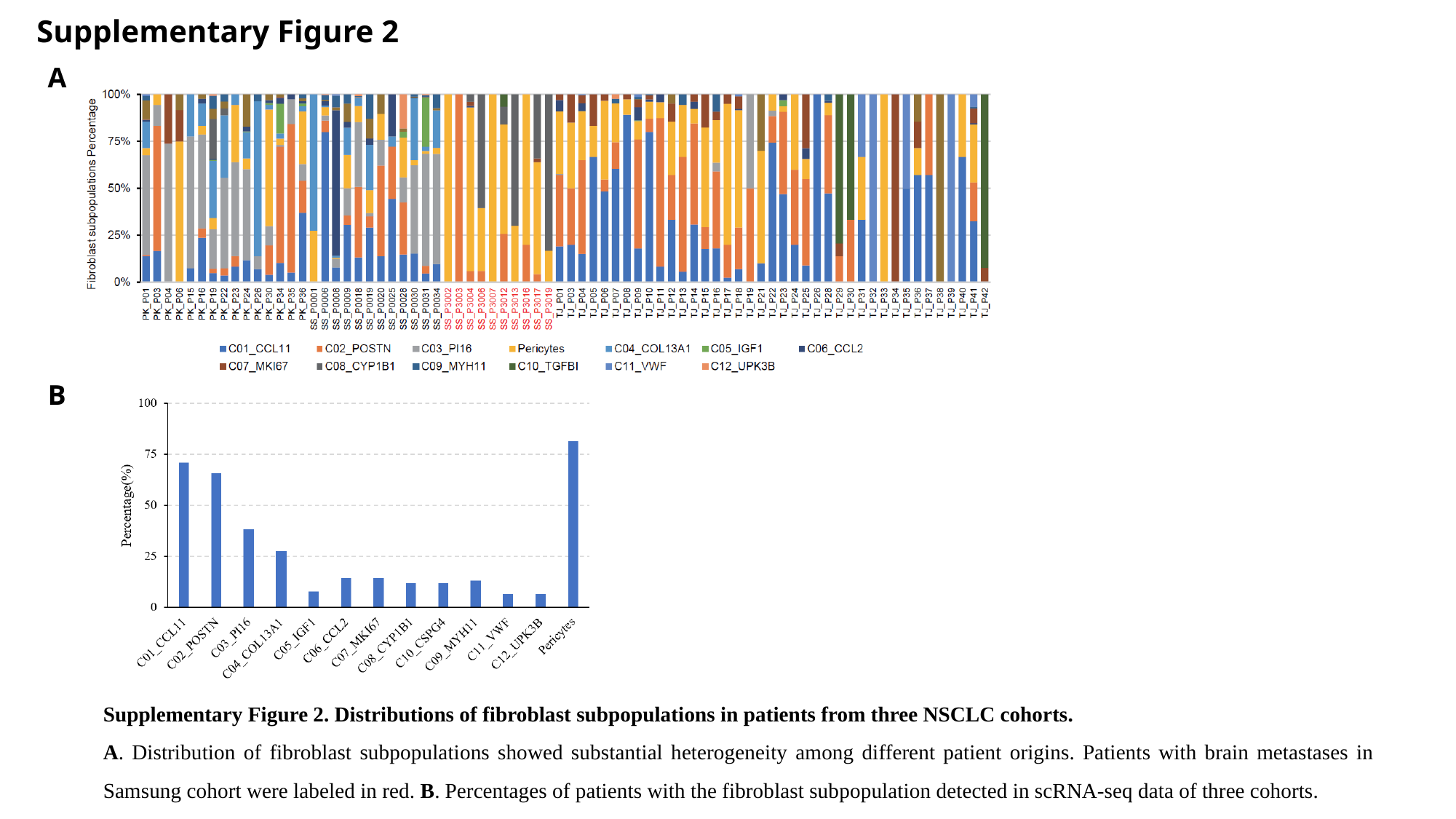

# Supplementary Figure 2
A
B
Supplementary Figure 2. Distributions of fibroblast subpopulations in patients from three NSCLC cohorts.
A. Distribution of fibroblast subpopulations showed substantial heterogeneity among different patient origins. Patients with brain metastases in Samsung cohort were labeled in red. B. Percentages of patients with the fibroblast subpopulation detected in scRNA-seq data of three cohorts.

## Slide 5
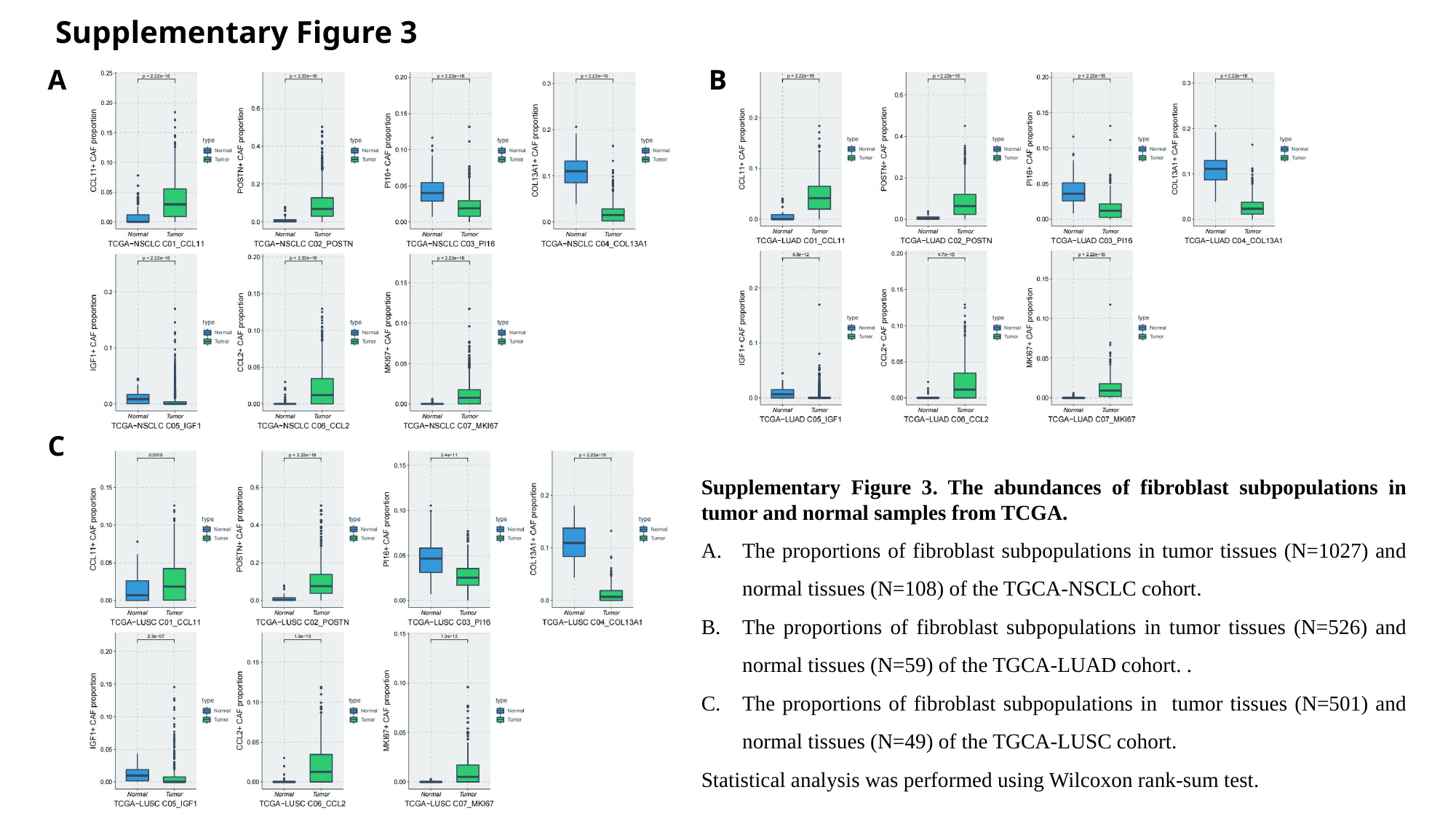

# Supplementary Figure 3
A
B
C
Supplementary Figure 3. The abundances of fibroblast subpopulations in tumor and normal samples from TCGA.
The proportions of fibroblast subpopulations in tumor tissues (N=1027) and normal tissues (N=108) of the TGCA-NSCLC cohort.
The proportions of fibroblast subpopulations in tumor tissues (N=526) and normal tissues (N=59) of the TGCA-LUAD cohort. .
The proportions of fibroblast subpopulations in tumor tissues (N=501) and normal tissues (N=49) of the TGCA-LUSC cohort.
Statistical analysis was performed using Wilcoxon rank-sum test.

## Slide 6
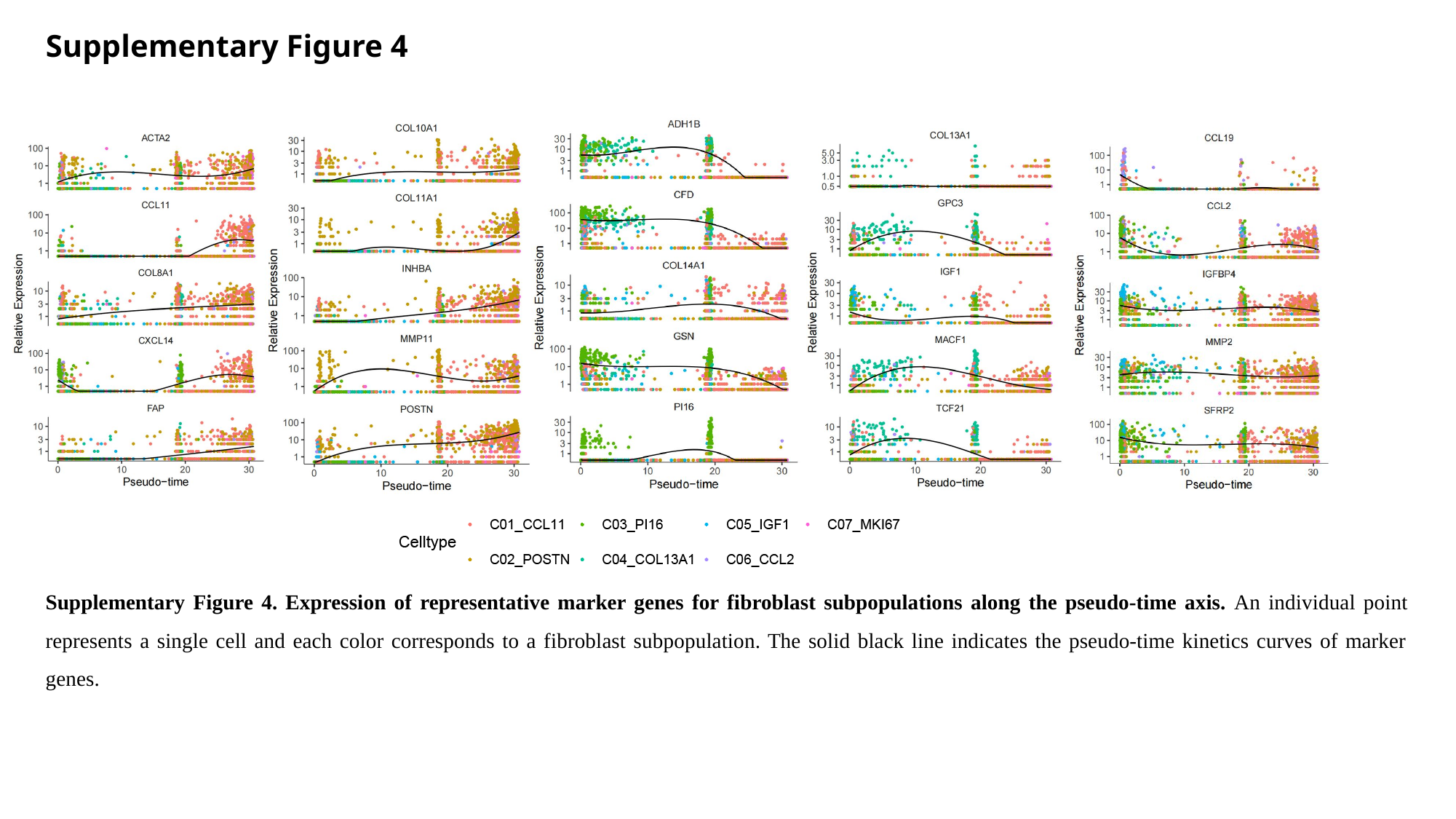

Supplementary Figure 4
Supplementary Figure 4. Expression of representative marker genes for fibroblast subpopulations along the pseudo-time axis. An individual point represents a single cell and each color corresponds to a fibroblast subpopulation. The solid black line indicates the pseudo-time kinetics curves of marker genes.

## Slide 7
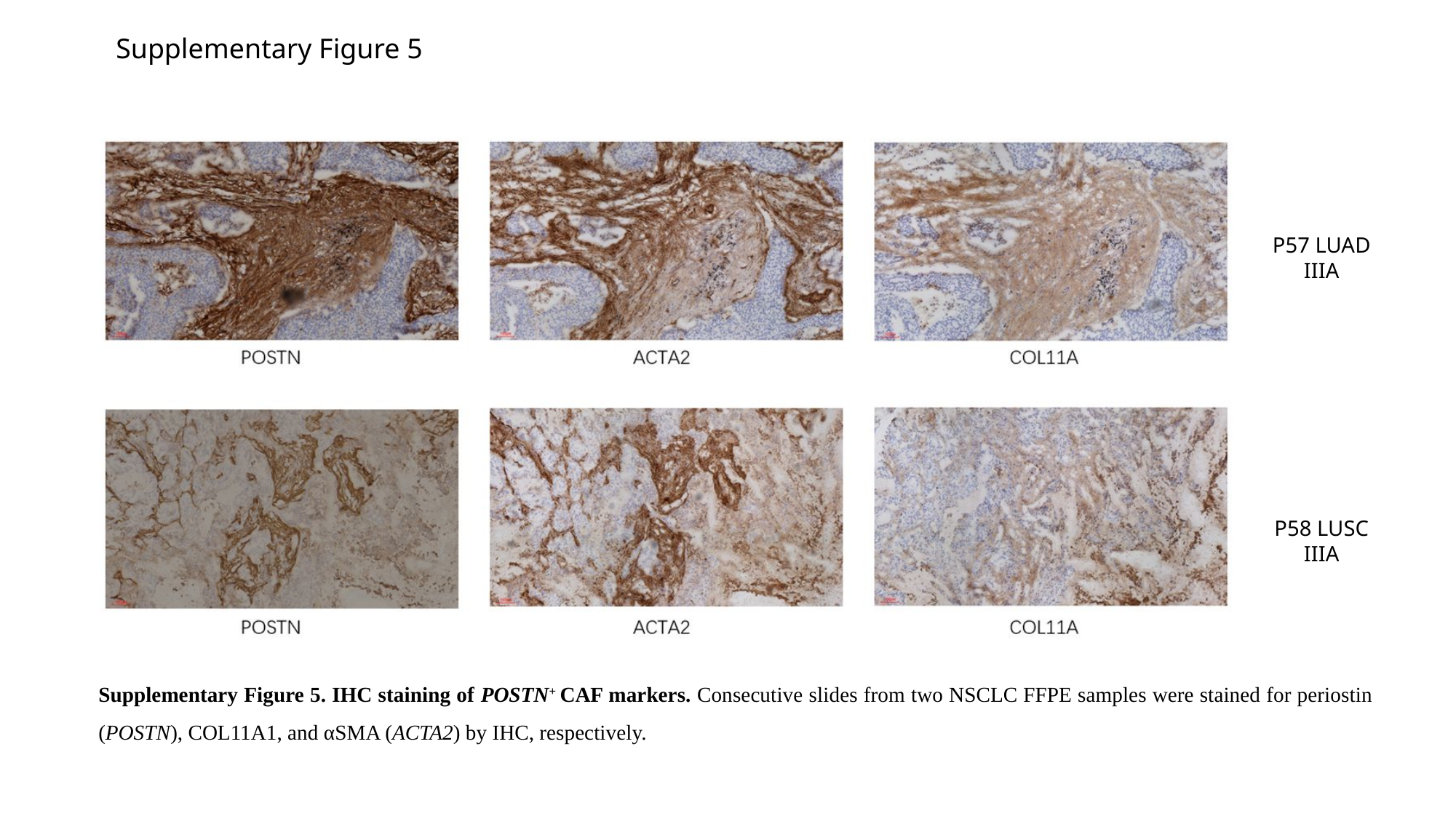

Supplementary Figure 5
P57 LUAD IIIA
P58 LUSC IIIA
Supplementary Figure 5. IHC staining of POSTN+ CAF markers. Consecutive slides from two NSCLC FFPE samples were stained for periostin (POSTN), COL11A1, and αSMA (ACTA2) by IHC, respectively.

## Slide 8
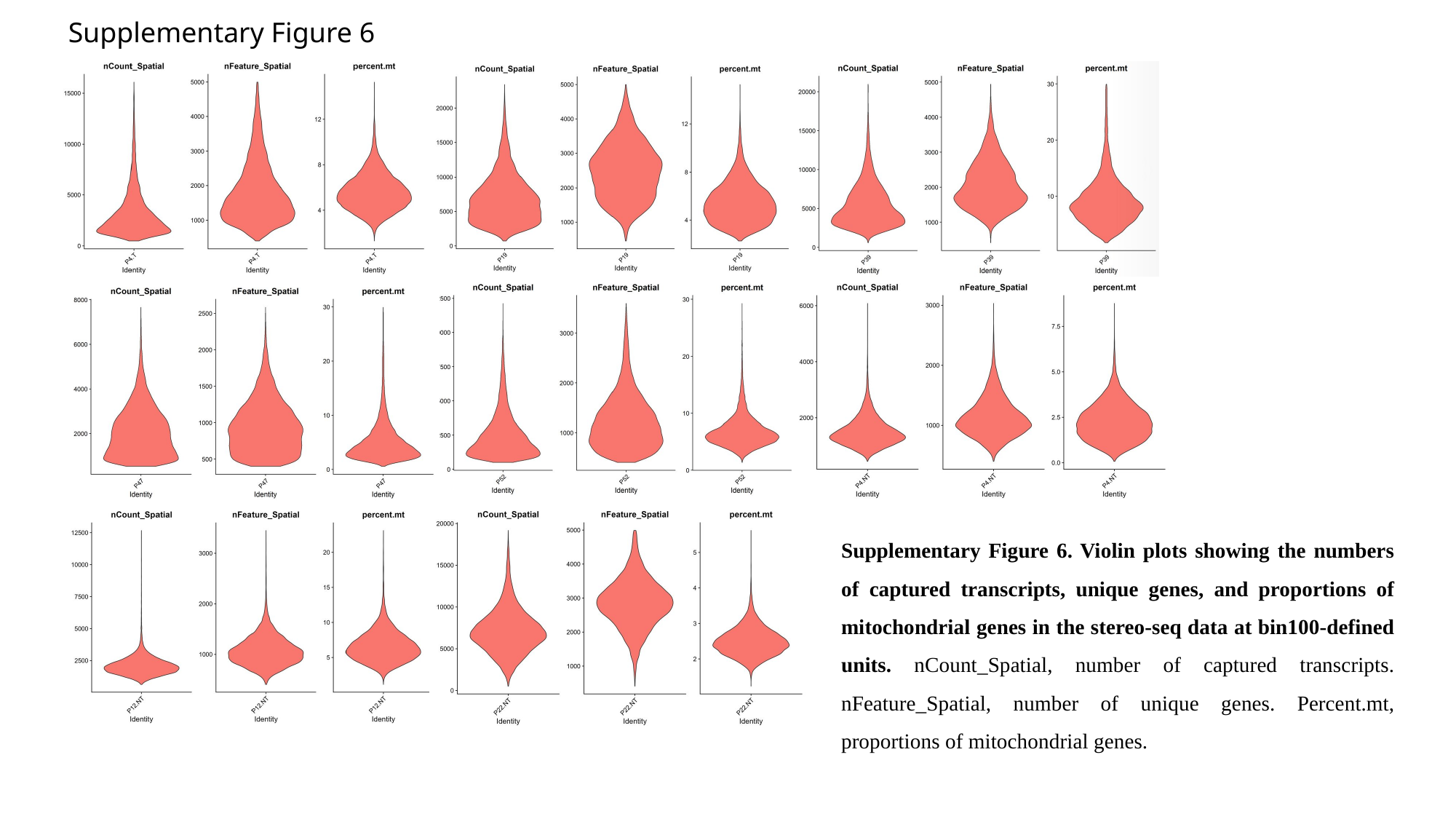

Supplementary Figure 6
Supplementary Figure 6. Violin plots showing the numbers of captured transcripts, unique genes, and proportions of mitochondrial genes in the stereo-seq data at bin100-defined units. nCount_Spatial, number of captured transcripts. nFeature_Spatial, number of unique genes. Percent.mt, proportions of mitochondrial genes.

## Slide 9
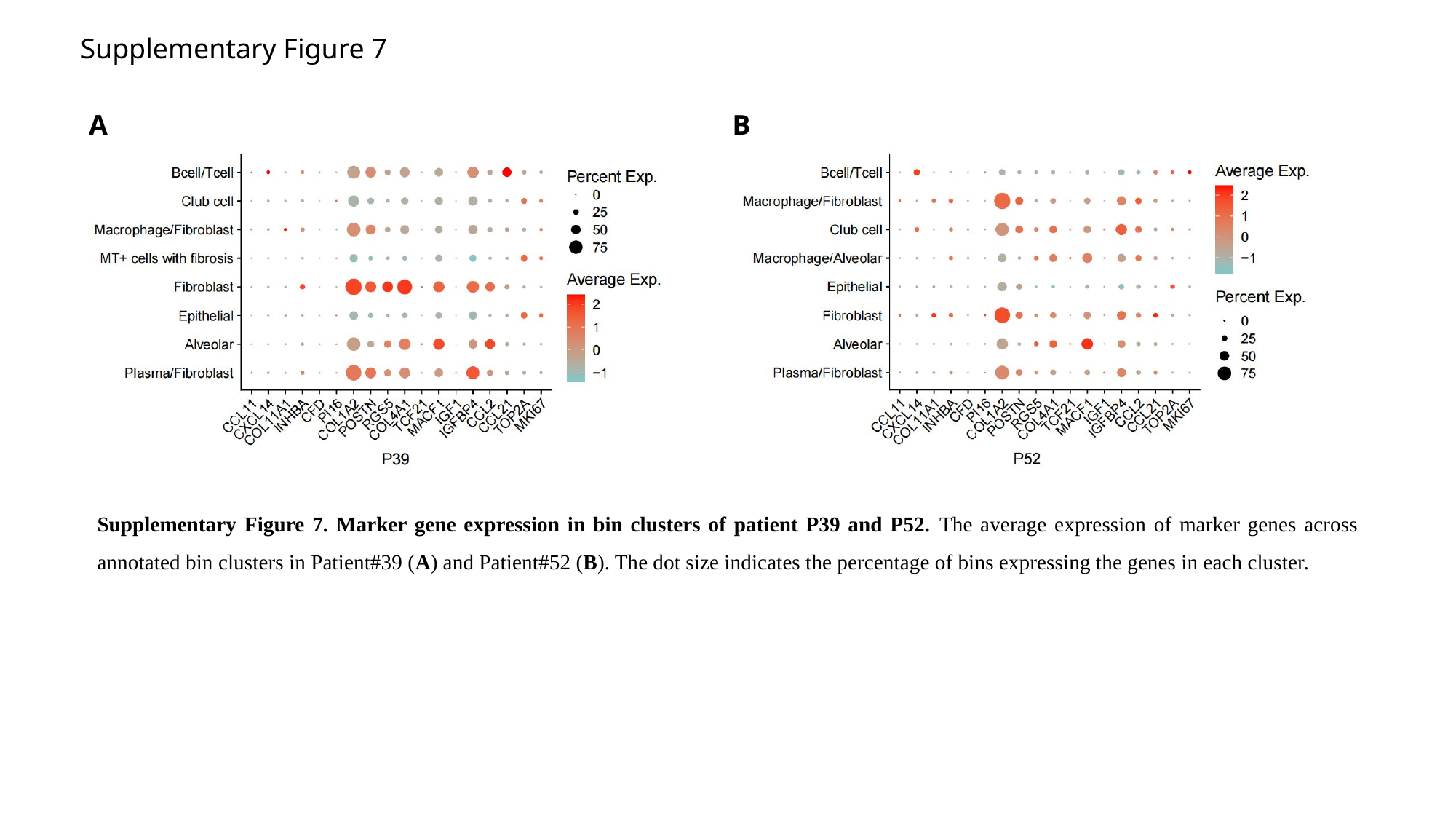

Supplementary Figure 7
A
B
Supplementary Figure 7. Marker gene expression in bin clusters of patient P39 and P52. The average expression of marker genes across annotated bin clusters in Patient#39 (A) and Patient#52 (B). The dot size indicates the percentage of bins expressing the genes in each cluster.

## Slide 10
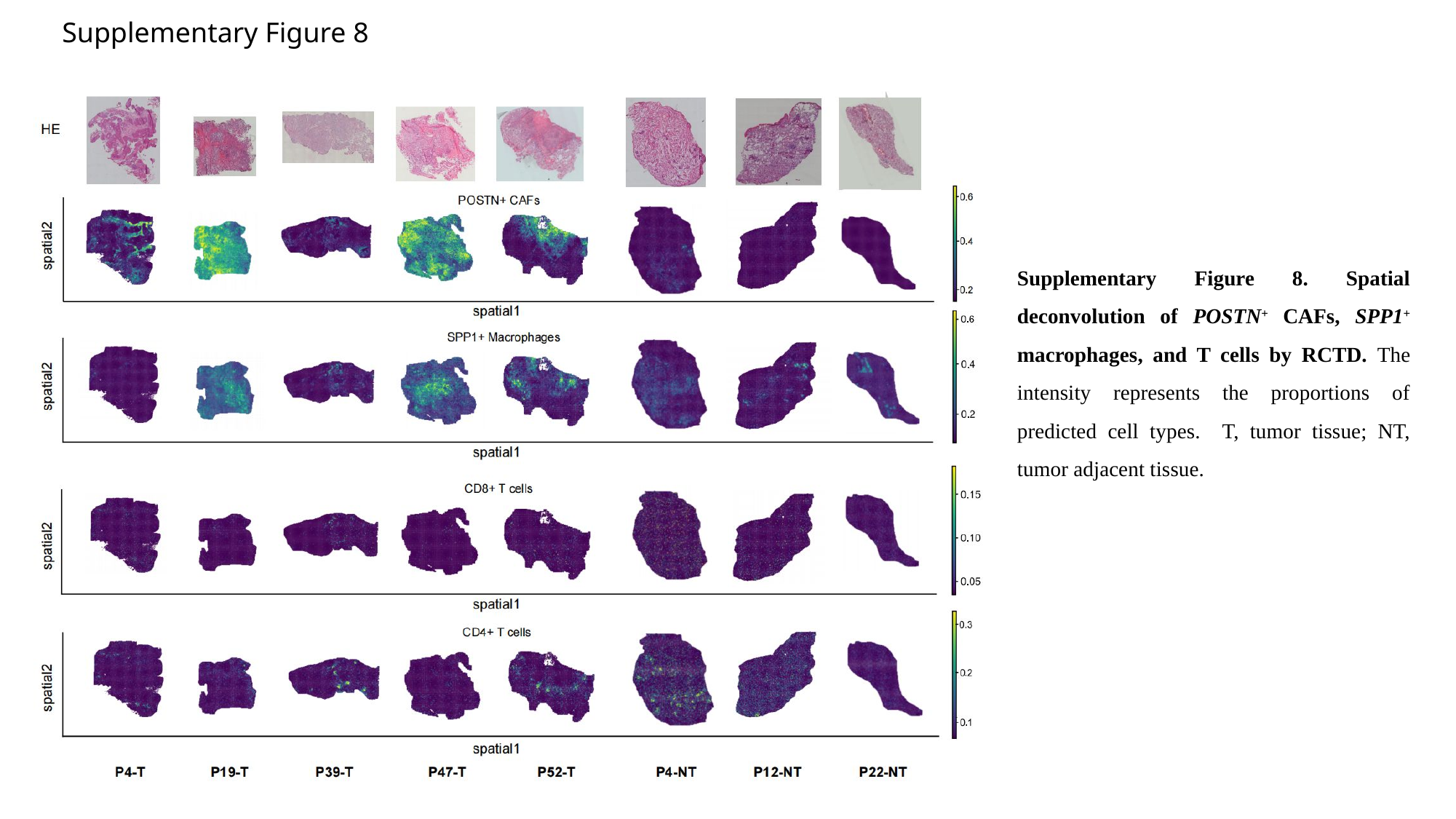

Supplementary Figure 8
Supplementary Figure 8. Spatial deconvolution of POSTN+ CAFs, SPP1+ macrophages, and T cells by RCTD. The intensity represents the proportions of predicted cell types. T, tumor tissue; NT, tumor adjacent tissue.

## Slide 11
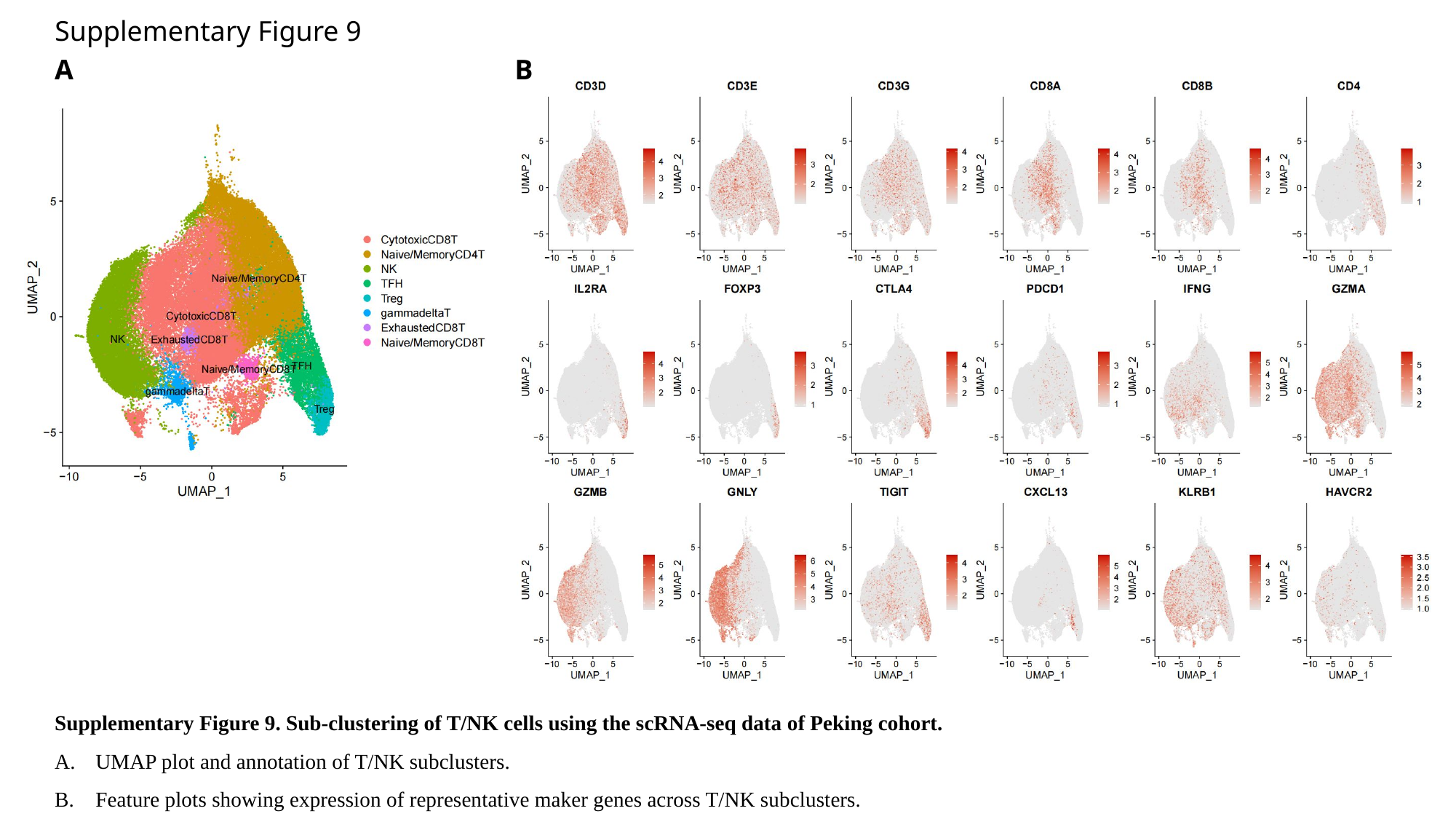

Supplementary Figure 9
A
B
Supplementary Figure 9. Sub-clustering of T/NK cells using the scRNA-seq data of Peking cohort.
UMAP plot and annotation of T/NK subclusters.
Feature plots showing expression of representative maker genes across T/NK subclusters.

## Slide 12
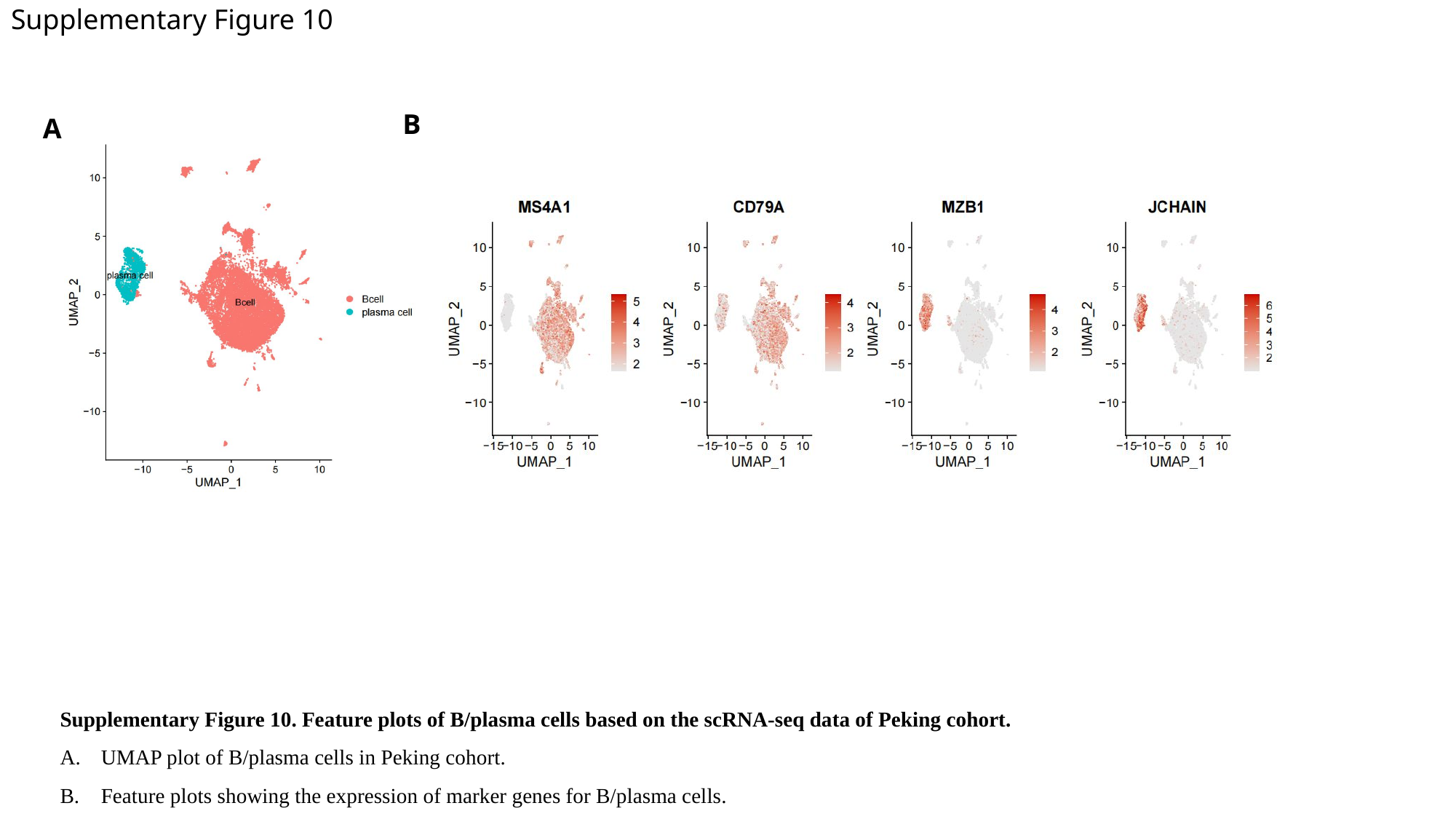

# Supplementary Figure 10
B
A
Supplementary Figure 10. Feature plots of B/plasma cells based on the scRNA-seq data of Peking cohort.
UMAP plot of B/plasma cells in Peking cohort.
Feature plots showing the expression of marker genes for B/plasma cells.

## Slide 13
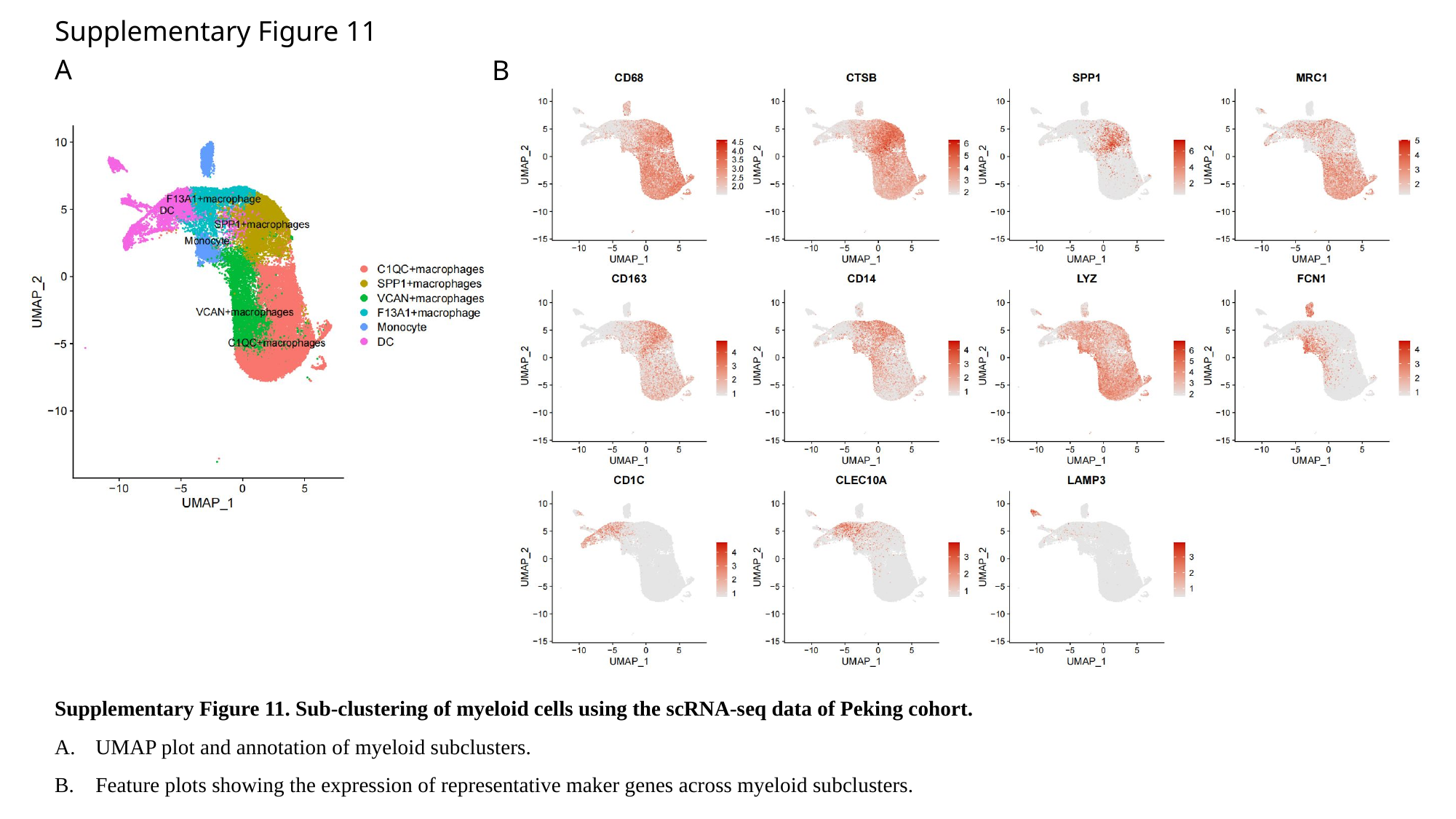

Supplementary Figure 11
A
B
Supplementary Figure 11. Sub-clustering of myeloid cells using the scRNA-seq data of Peking cohort.
UMAP plot and annotation of myeloid subclusters.
Feature plots showing the expression of representative maker genes across myeloid subclusters.

## Slide 14
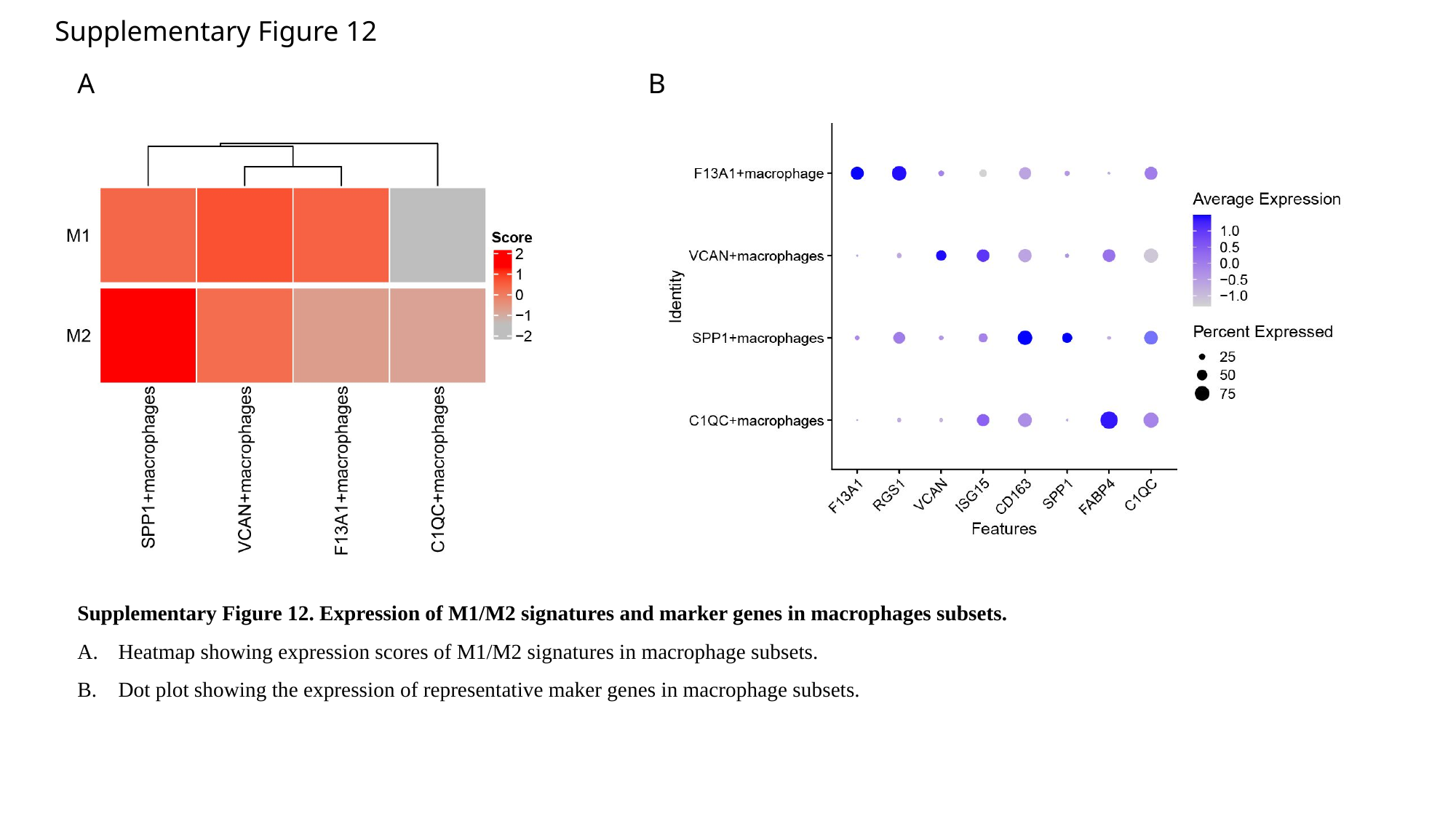

Supplementary Figure 12
A
B
Supplementary Figure 12. Expression of M1/M2 signatures and marker genes in macrophages subsets.
Heatmap showing expression scores of M1/M2 signatures in macrophage subsets.
Dot plot showing the expression of representative maker genes in macrophage subsets.

## Slide 15
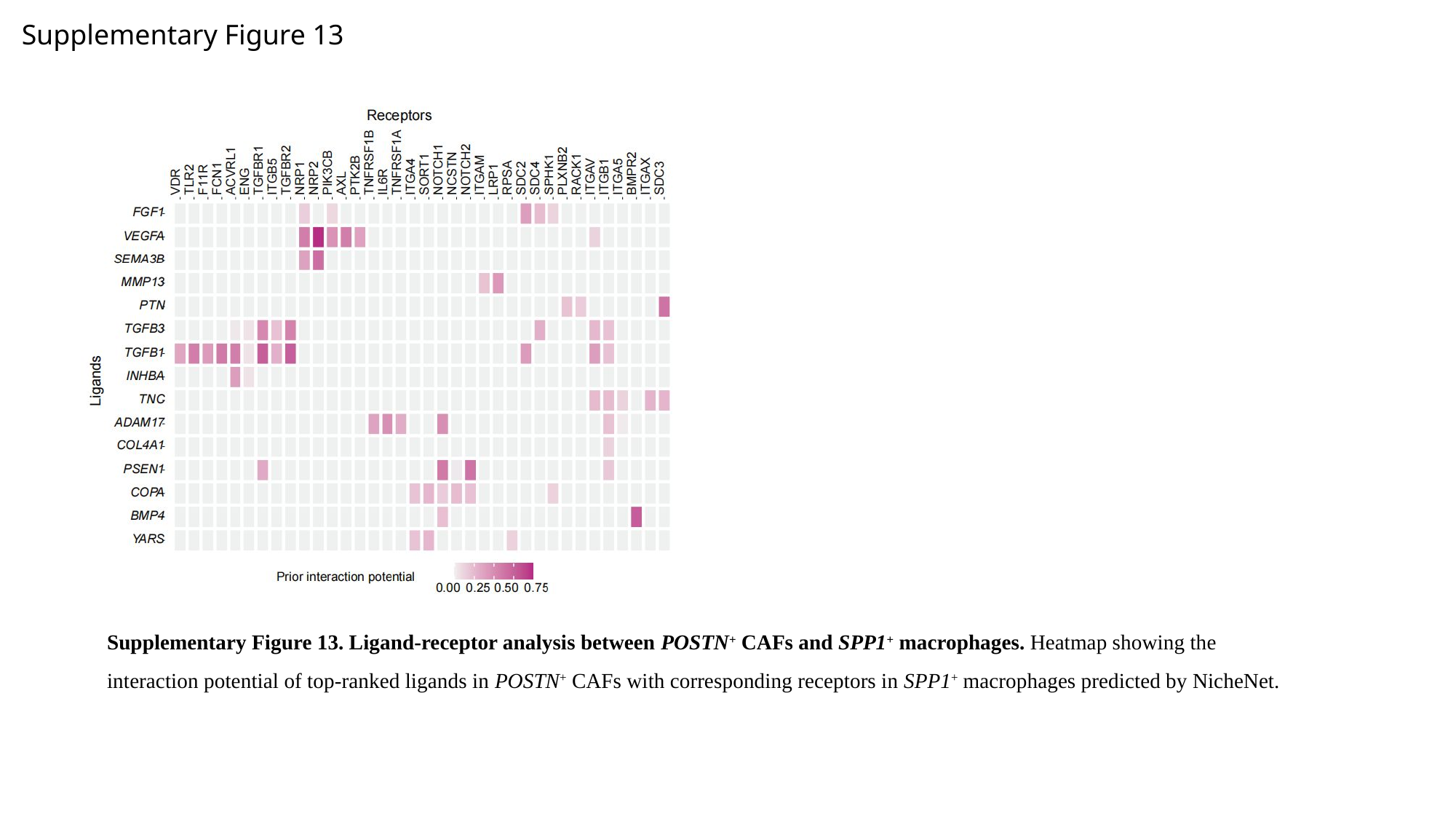

# Supplementary Figure 13
Supplementary Figure 13. Ligand-receptor analysis between POSTN+ CAFs and SPP1+ macrophages. Heatmap showing the interaction potential of top-ranked ligands in POSTN+ CAFs with corresponding receptors in SPP1+ macrophages predicted by NicheNet.

## Slide 16
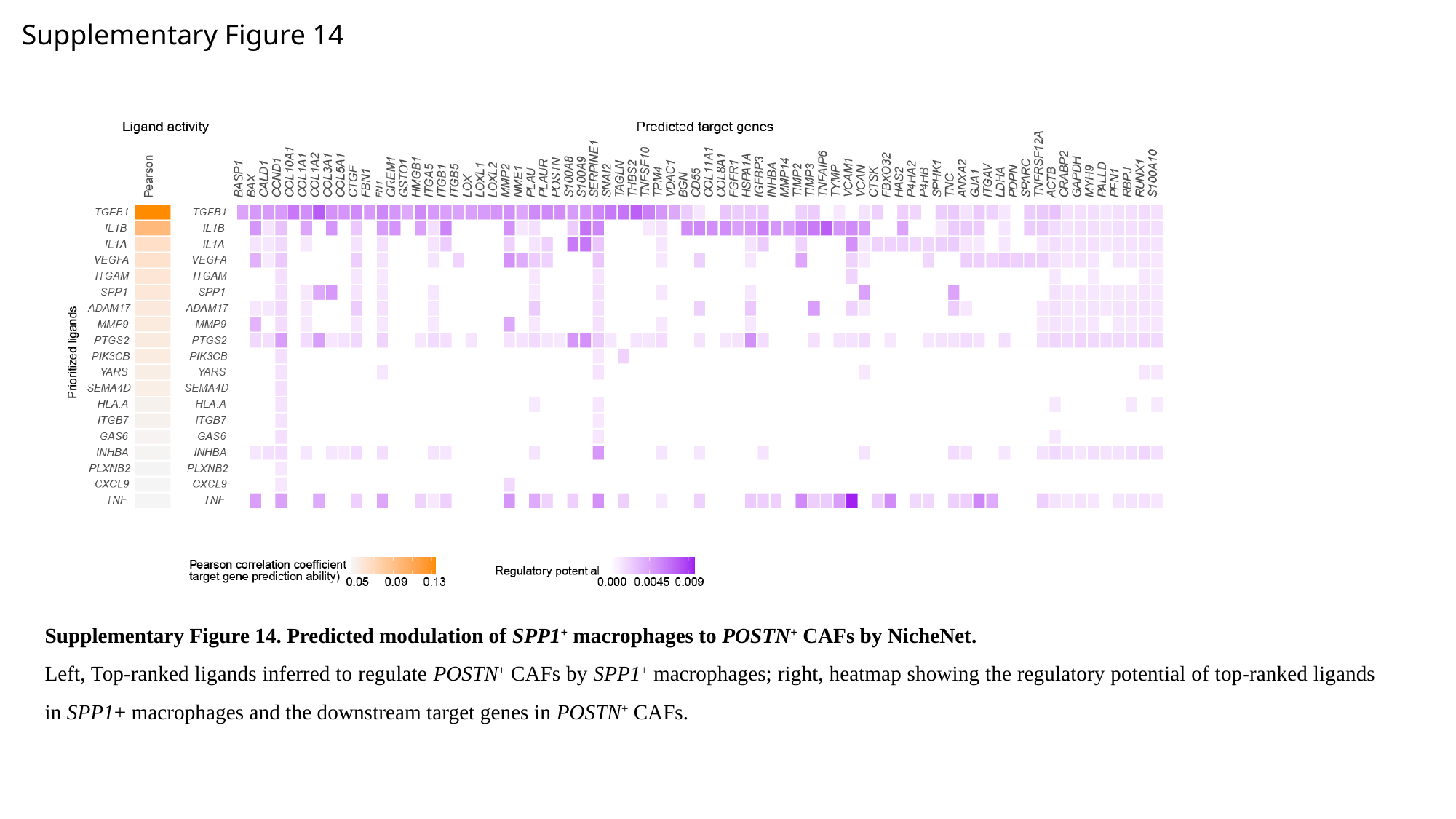

# Supplementary Figure 14
Supplementary Figure 14. Predicted modulation of SPP1+ macrophages to POSTN+ CAFs by NicheNet.
Left, Top-ranked ligands inferred to regulate POSTN+ CAFs by SPP1+ macrophages; right, heatmap showing the regulatory potential of top-ranked ligands in SPP1+ macrophages and the downstream target genes in POSTN+ CAFs.

## Slide 17
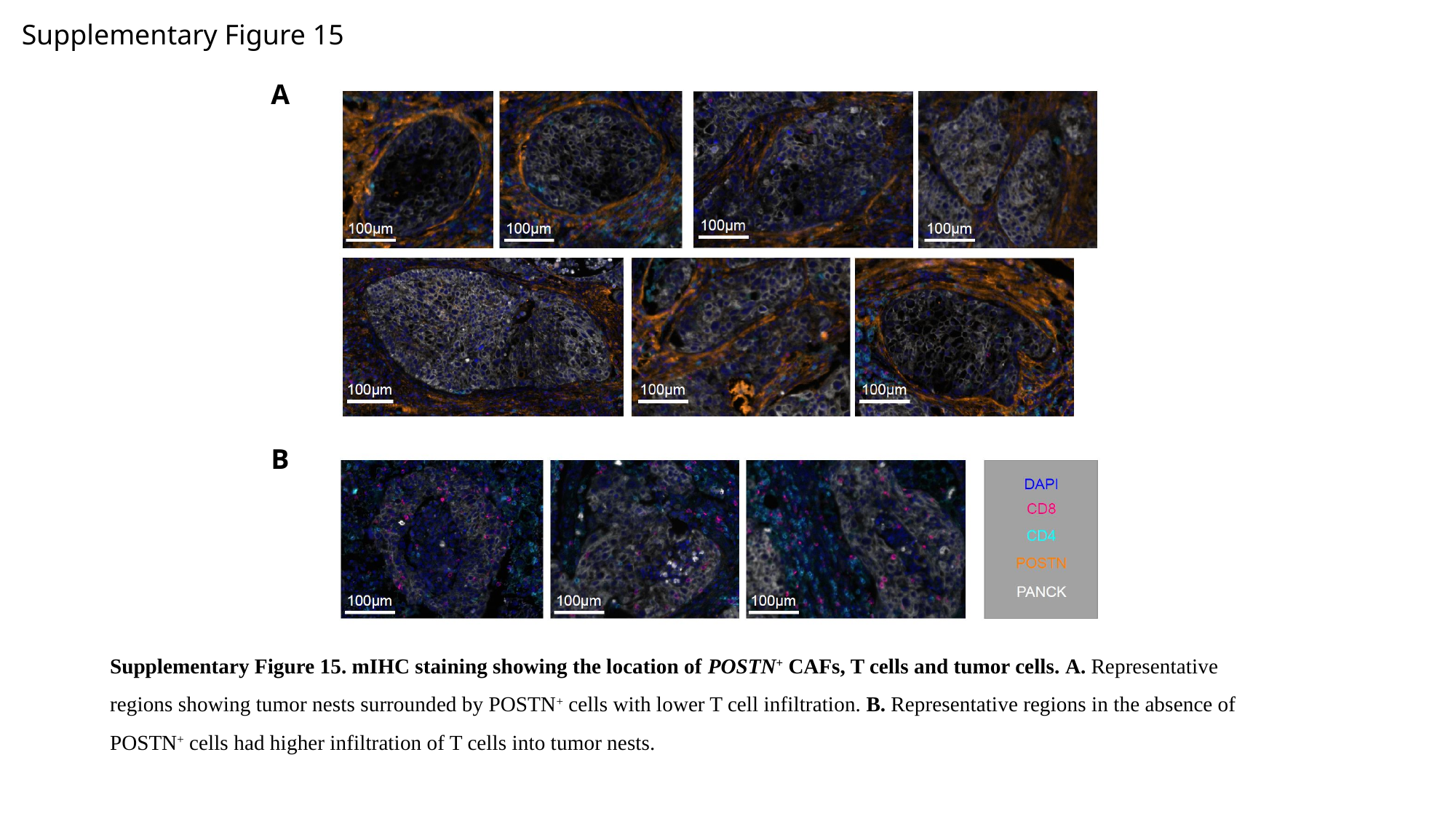

Supplementary Figure 15
A
B
Supplementary Figure 15. mIHC staining showing the location of POSTN+ CAFs, T cells and tumor cells. A. Representative regions showing tumor nests surrounded by POSTN+ cells with lower T cell infiltration. B. Representative regions in the absence of POSTN+ cells had higher infiltration of T cells into tumor nests.

## Slide 18
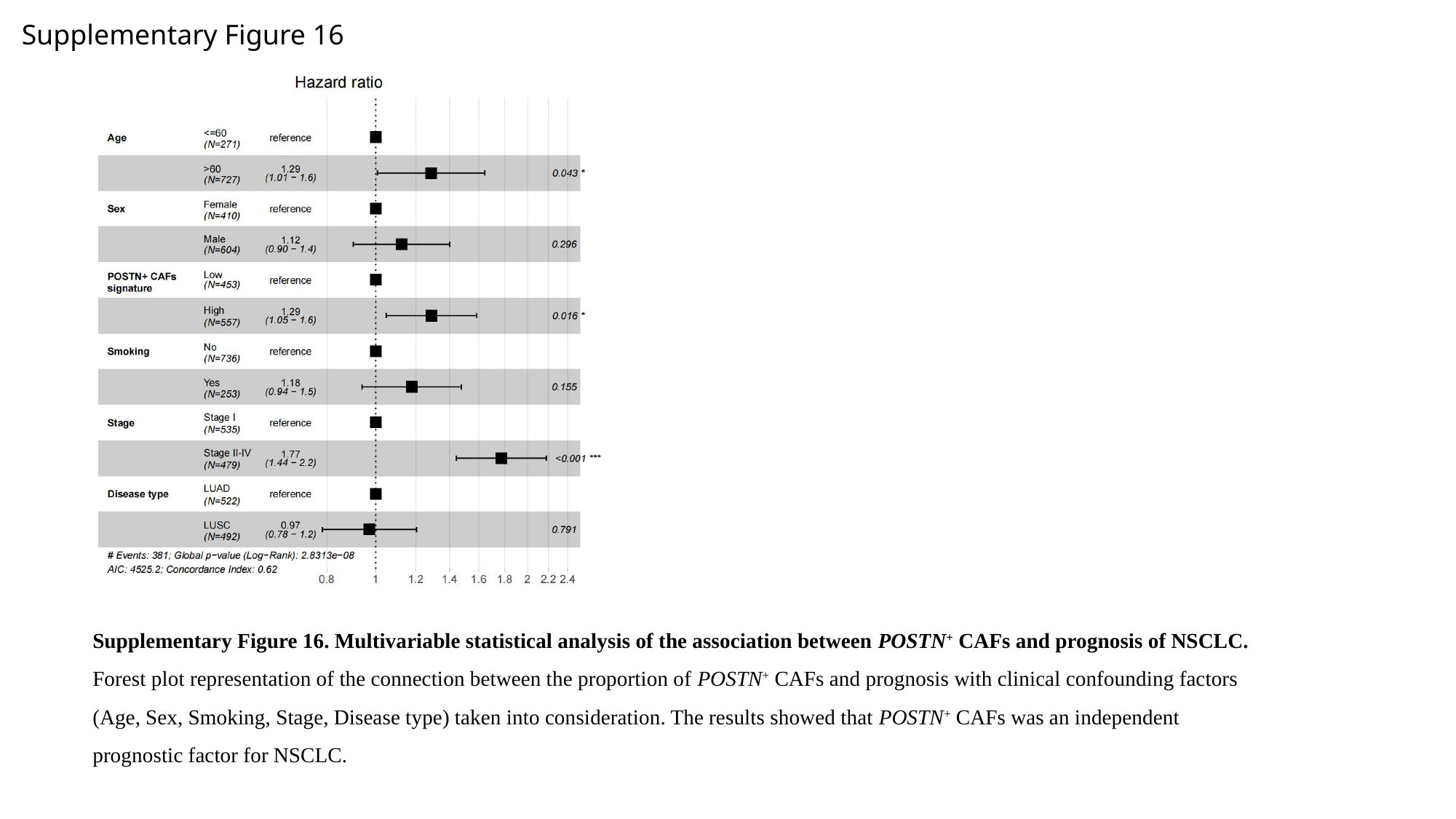

Supplementary Figure 16
Supplementary Figure 16. Multivariable statistical analysis of the association between POSTN+ CAFs and prognosis of NSCLC. Forest plot representation of the connection between the proportion of POSTN+ CAFs and prognosis with clinical confounding factors (Age, Sex, Smoking, Stage, Disease type) taken into consideration. The results showed that POSTN+ CAFs was an independent prognostic factor for NSCLC.
